# Occurrence and Definitions of Intra and Postoperative Complications Related to Laparoscopy in Equids: A Scoping Review

**DOI:** 10.3390/vetsci9100577

**Published:** 2022-10-17

**Authors:** Anna Cerullo, Marco Gandini, Gessica Giusto

**Affiliations:** Department of Veterinary Sciences, University of Turin, Largo Paolo Braccini 2-5, 10095 Grugliasco, Italy

**Keywords:** horse, laparoscopy, mini-invasive surgery, intra-operative complications, postoperative complications, scoping review

## Abstract

**Simple Summary:**

Laparoscopy and laparoscopic-assisted procedures in equines are nowadays common procedures with several advantages compared to laparotomy. However, despite the numerous benefits of minimally invasive surgery, there can be surgical complications which could have important welfare and economic consequences. Nevertheless, standard definitions of intra and postoperative complications are rarely reported and there is a lack of standard criteria to define and classify complications, limiting comparison of complication rates between studies. Thus, there is a need for implementation of rigorous criteria for defining complications and for greater numbers of research study with high quality of evidence. Adoption of classification systems and standard definitions would help surgeons to have a complete picture of the efficacy of a procedure or treatment and it is also essential to allow comparisons between studies, centers or time periods.

**Abstract:**

Laparoscopy is a common approach in equine surgery and has the advantage of improved visibility and diagnostic accuracy, decreased morbidity and hospitalization time. However, despite the numerous benefits, there can be intraoperative and postoperative complications which could have important welfare and economic consequences. The aim of this study was to perform a scoping review to identify current evidence on the occurrence, definition and classification of intra and postoperative complications in equine laparoscopy. A scoping review was conducted in scientific databases. Peer-reviewed scientific articles in the English language on laparoscopy in equids between 1992 and 2022 were included. Data on the study method, sample size, surgical procedure, intra and postoperative complications were extracted and charted. One hundred sixty-four articles met the final inclusion criteria. A definition of “intraoperative complication” was given in one study. Difference between “minor” or “major” intraoperative complications were reported in 12 articles and between “minor” or “major” postoperative complications in 22 articles. A total of 22 intraoperative and 34 postoperative complications were described. The most reported intraoperative complication was hemorrhage from ovary or mesovary (12.7%), while the most reported postoperative complications were incisional complications (64.2%) and postoperative pain (32.7%). There is a need for implementation of criteria for defining complications. The adoption of classification systems and standard definitions would help surgeons to make decisions about the most appropriate treatment, and it is also essential to allow comparisons between research results.

## 1. Introduction

The laparoscopic approach to equine abdomen has numerous benefits such as the improved visibility of organs, with the advantage of being a minimally invasive procedure. Furthermore, laparoscopy is often a standing procedure, and this avoids the risk of general anesthesia and allows a rapid postoperative recovery, with decreased patient stress and postoperative pain, reduced morbidity and hospitalization stay [1,2,3,4]. Laparoscopy and laparoscopic-assisted procedures are increasingly used and in some cases are replacing open surgical approaches. There is a wide use of laparoscopic surgery to perform gonadectomy, biopsy or tumor removal. However, despite the numerous benefits of minimally invasive surgery, intraoperative and postoperative complications could have important welfare and economic consequences. Equine surgeons need a detailed analysis of complications that could arise during and after laparoscopic surgeries to improve surgical techniques and to fully evaluate the success of treatment.

Based on the increase in the number of publications related to equine laparoscopy in recent years, there is a need to assess the quality of studies in the literature and to have an updated review of complications most frequently associated with laparoscopy and laparoscopic-assisted procedures. It is also important to appropriately evaluate adverse events and to facilitate clear communication of research results to owners and referring vets [5,6,7,8,9,10]. In 1992, the first classification system for postoperative complications in human medicine was proposed. Since then, several grading systems for postoperative complications have been developed to report clinical research findings into human surgical practice [7,8,11,12]. Several grading systems for perioperative outcome were proposed in veterinary medicine, mostly for small animals [9,13,14,15,16,17,18]. Rigorous and standardized classification criteria have never been proposed for equine surgery. This lack of clarity over intra and postoperative complication definitions and classifications was a good prerequisite for the realization of this scoping review.

A scoping review aims to map the existing search literature on a specific topic and does not perform any critical analysis of the studies identified [19]. Scoping reviews investigate research conduct and reveal knowledge gaps in a body of literature through methodological database searching [20,21]. A scoping review of a body of literature can be particularly useful when the topic has not yet been extensively reviewed or is of a complex or heterogeneous nature [19]. Therefore, this type of review can be used to investigate research and identify knowledge gaps regarding surgical complications in studies on equine laparoscopy. This study reviewed the literature on laparoscopy and laparoscopic-assisted surgeries reported in clinical research studies of client-owned or health horses over the last 30 years. One of the aims of this study was to evaluate how often each intra and postoperative complication was reported in the literature, how often the term “complication” was defined and how often complications were classified and graded for severity. Furthermore, this study summarized the surgical complications reported and the definitions used for each complication.

## 2. Materials and Methods

### 2.1. Protocol and Registration 

Two researchers conducted this in duplicate according to the Preferred Reporting Items for Systemic reviews and Meta-Analyses extension for Scoping Reviews (PRISMA-ScR) [21]. The protocol was registered at the OSF Registries and can be downloaded at the following link: https://osf.io/efzcp. All authors helped create the database search strategy and any disagreements between the two researchers were resolved by a third independent reviewer.

### 2.2. Eligibility Criteria

Inclusion criteria included peer-reviewed English-language scientific articles on laparoscopy or laparoscopic-assisted procedures in client-owned or healthy research horses, donkeys and mules between 1992 and 2022. Exclusion criteria included studies that did not provide information regarding occurrence of postoperative complications, review articles, editorials, studies involving other animals than equids and ex vivo studies. Studies that provided information regarding occurrence of intraoperative complications but did not provide any information relating to short- or long-term postoperative complications were also excluded.

### 2.3. Information Sources and Search Strategy

The search for potentially eligible articles started in July 2022 in the CAB, Web of Science, Scopus, and PubMed databases. Search combinations were constructed from the following components using a PCC (population, concept, and context) search strategy according to the JBI guidelines (https://jbi-global-wiki.refined.site/space/MANUAL/4687737/11.2.2+Developing+the+title+and+question-last accessed on 29 August 2022).

Population: Horses, donkeys, mules (equids).

Concept: Complications, adverse events, sequelae, failure to cure, technical failure and disease progression.

Context: Laparoscopy or laparoscopic-assisted procedure.

The resulting search string was as follows:

- (Horse OR equine OR mule OR donkey) AND (laparoscopy OR laparoscopies);
- Years = “1992–2022”;
- Language = “English”;
- Publication type = “journal article”.

The details of the search strings for each database are detailed in Supplementary material 1. The resulting references were downloaded and managed with Clarivate Endnote Online (https://access.clarivate.com/login?app=endnote- last accessed on 29 August 2022).

### 2.4. Selection of Sources of Evidence

Publications that were duplicates were immediately excluded using the relevant EndNote Online tool. At least two reviewers (M.G. and A.C.) independently and blindly screened each title, abstract and full text, as required, to select studies’ eligibility by two investigators (M.G. and A.C.) based on the titles, abstracts, and full texts. Any disagreement between the two researchers was resolved by a third researcher (G.G.).

### 2.5. Data Charting Process 

The full-text studies were independently analyzed by two researchers (M.G., A.C.), and relevant data were charted. Data on the study characteristics were extracted under the following headings: Complete article citation, type of study, location, species, sample size, type of procedure, apparatus involved, surgical purpose, use or not of CO_2_ insufflation. Complications were classified as intraoperative or postoperative using definitions accepted for human surgery. In human surgery, intraoperative complication is defined as any deviation from the ideal intraoperative course occurring between skin incision and skin closure [10], while surgical complications in general is defined as any deviation from the normal postoperative course [7,8]. “Perioperative” category was not used because of inconsistency in its meaning within the literature [4,17]. Data on intra and postoperative complications were extracted and reported. Follow-up was divided in short- or long-term according to the time reported in each article if it was specifically reported. For each article, the following data were also collected: whether the term complication was explicitly defined in the study, whether the definition for each complication was provided, and whether the complications were considered “major” or “minor”. The relative incidence of complications was calculated as a percentage of cases with a complication on the total number of cases described in the articles which reported that given complication. The cumulative incidence was calculated as a percentage of cases with a given complications on the total number of cases in the articles charted. Summary statistics were calculated, and data were reported as medians (range). For the purpose of this study, we included in the definition of “postoperative pain” articles reporting “colic”, “signs of discomfort” and “postoperative pain” and in the definition of “incisional complications” articles reporting “oedema”, “subcutaneous emphysema”, “seroma”, “surgical site infection”, “wound dehiscence”, “skin incision inflammation”, “incisional complications”, “parietal pain” and “depression of the musculature”. These two most common postoperative complications (postoperative pain and incisional complications) were also compared based on type of approach (standing, recumbent or both), apparatus involved (gastrointestinal, urogenital, both or other) and use or not of CO_2_ insufflation through a Chi-square test. Statistical significance will be set with *p* < 0.05, the evidence was presented in narrative form and in tables and charts.

## 3. Results

### 3.1. Selection of Sources of Evidence 

**Figure 1:**
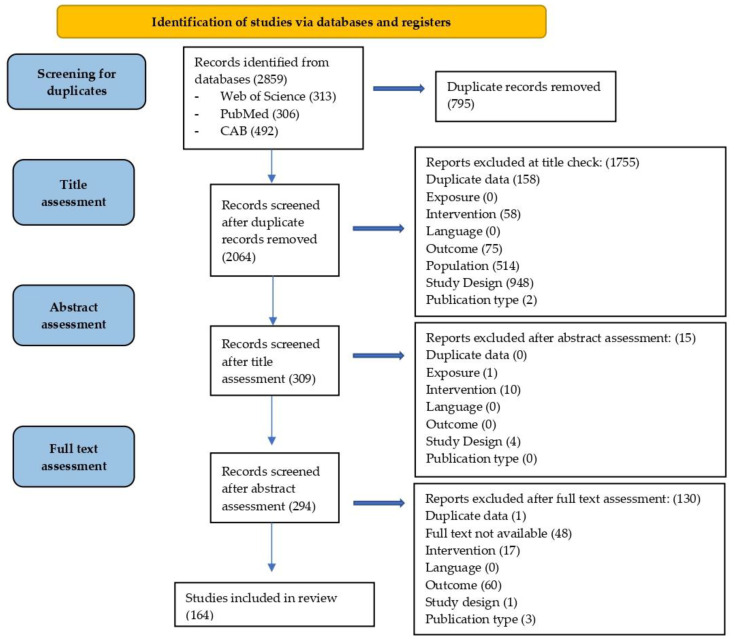
Study selection flow chart used to identify studies on intra- and postoperative complications related to laparoscopy in equids.

A total of 2859 studies were identified in the initial database search. The flowchart of publication search and assessment, as outlined in the selection of evidence sources, is reported in Figure 1. After title and abstract review, 294 full-text articles were assessed, and 164 articles met inclusion criteria reporting data of 2321 equids.

### 3.2. Characteristics of the Sources of Evidence 

**Figure 2:**
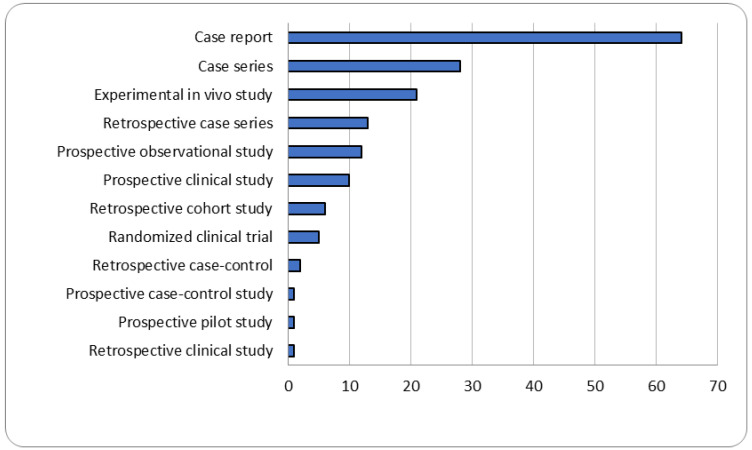
Publication types on surgical complications related to laparoscopy in equids throughout the last 30 years.

**Figure 3:**
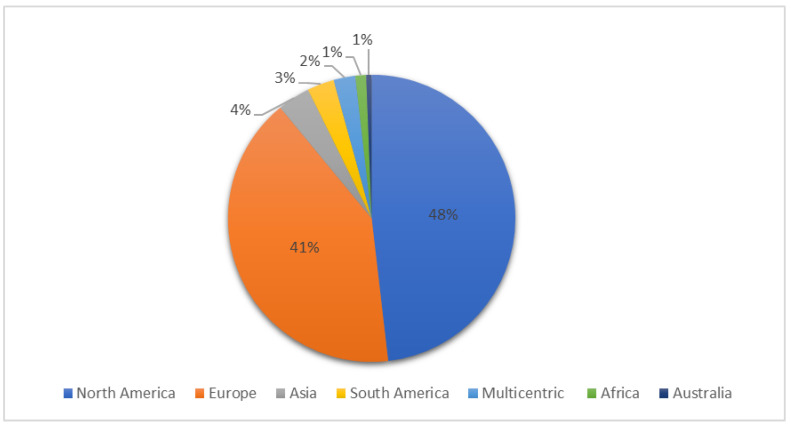
Location of studies on surgical complications related to laparoscopy in equids.

The most frequent types of study design were retrospective case reports (64/164) and case series (28/164), followed by experimental in vivo studies (21/164), retrospective case series (13/164), prospective observational studies (12/164), prospective clinical studies (10/164), retrospective cohort studies (6/164), randomized clinical trial (5/164), retrospective case–control study (2/164), retrospective clinical study (1/164), prospective pilot study (1/164) and prospective case–control study (1/164). The different types of publication throughout the last 30 years are reported in Figure 2. Most of the studies were conducted in North America and Europe (Figure 3). The median sample size was 6 animals (range 1–241), leading to a total of 2321 equids. A total of 99 of 164 (60.3%) studies reported surgical procedure on urogenital apparatus, 30 of 164 (18.2%) on gastrointestinal apparatus and 1/164 on both (0.6%). Additionally, 34 out of 164 articles were focused on procedures regarding other organs and apparata (20.7%). A standing laparoscopic approach was described in 126 (76.8%) studies, a recumbent approach was described in 32 (19.5%) studies, and both approaches were reported in 6 studies (3.6%). Most articles (35.3%) reported laparoscopic approach for gonadectomy, followed by 23 of 164 articles (14%) reported space or ring closure procedure, 13 of 164 (7.9%) hernioplasty or herniorrhaphy and 12 of 164 each (7.3%) for tumor removal or organ rupture repair. The complete data charting of all the sources is reported in Supplementary material 2.

### 3.3. Results of Individual Sources of Evidence 

Only in one article there was a definition for intraoperative complication. Intraoperative complications were characterized according to the severity in 12/164 studies and postoperative complications in 22/164 studies, which described them as “major” or “minor” complications. One article reported the term “technical error” and two articles the term “sequela” without defining them, thus using the term as a synonymous of “complications”. Furthermore, no article used or mentioned a classification of complications from either human or veterinary surgery. A total of 53 out of 164 articles (32.3%) reported intraoperative complications, and a total of 21 intraoperative complications were described in 145/2321 equids (cumulative incidence of intraoperative complications was 6.25%). Only 1 article reported a definition for intraoperative complication, which was defined as “a situation in surgery that was not part of the initial surgical plan” [22], and in 2/164 articles, intraoperative complications reported (signs of discomfort and poor portal placement) were also defined. The most commonly reported intraoperative complications was hemorrhage from the ovary or mesovary (21/164, 12.7%) followed by accidental splenic injury (8/164, 4.8%), parietal hemorrhage (7/164, 4.2%), ovary or testis dropping (6/164, 3.6%), signs of discomfort (5/164, 3%), poor visualization (4/164, 2.4%), accidental intestinal injury (3/164, 1.8%) and accidental thorax penetration (3/164, 1.8%). Complete list of intraoperative complications is reported in Table 1 and list of definitions is reported in Supplementary material 3 (Tables S1 and S2). Relative and cumulative incidence of intraoperative complications are reported in Table 2.

Incidence of hemorrhage from mesovary was not different between various methods of hemostasis (vessel sealing devices, ligatures and surgical staplers) or between different time periods (Tables S3 and S4).

Out of 164 articles (48.7%), 80 reported a total of 34 postoperative complications in 824/2321 equids (cumulative incidence of postoperative complications was 35.5%). The most commonly reported postoperative complications were incisional complications (106/164, 64.2%) followed by postoperative pain (54/164, 32.7%), fever (25/164 15.1%), tachycardia or cardiac failure (11/164, 6.6%), anorexia (7/164, 4.2%), depression (6/164, 3.6%) and diarrhea (6/164, 3.6%). Complete list of postoperative complications is reported in Table 3. Multiple definitions of these complications were reported. Nine different definitions were provided for signs of discomfort and colic in 11 articles, 7 definitions for incisional complications in 8 articles, 2 for piroplasmosis in 2 articles, 1 for phlebitis in 1 article and 1 for decrease in fecal output in 1 article. Complete list of definitions is reported in Supplementary material 3 (Tables S5–S13). Relative and cumulative incidence of postoperative complications are reported in Table 4.

**Figure 4:**
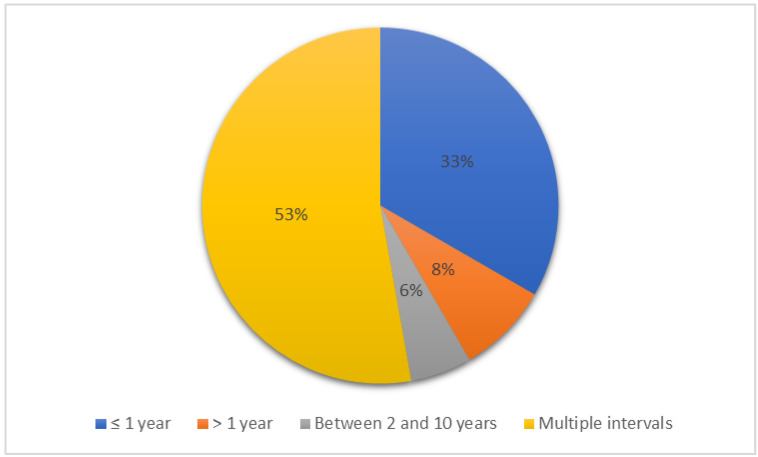
Time frames for long-term follow up reported in studies on surgical complications related to laparoscopy in equids.

The follow-up result was reported in 156/164 articles (95.1%) but only in 38 out of 156 the follow-up was clearly reported as short and long-term follow-up. Most studies (34/38 articles) considered short-term follow-up as the time between surgery and hospital discharge. The short-term follow-up period was defined as a time interval of 2 weeks after surgery in 2/38 articles, 14, 30 and 60 days after surgery in 1 article each and 6–8 weeks in 1 article. Among 38 articles, the long-term follow-up was reported in a time interval < 1 year after surgery in 12 studies, ≥ 1 year in 3 studies, between 2 and 10 years in 2 studies and in multiple long-term intervals in 19 studies (Figure 4).

Incisional complications were significantly (*p* < 0.00001) more common when both standing and recumbent approaches were used (57.1%), than when only recumbent (35.9%) or only standing approaches were used (20.1%). Furthermore, they were significantly (*p* < 0.00001) more common when gastrointestinal tract was involved in the procedure (50.7%) than urogenital (13.5%) or other organs and apparata (37.6%). The incidence of incisional complications was higher when CO_2_ insufflation was used (33.6% with insufflation, 20.3% no CO_2_ insufflation, *p* = 0.00003). Postoperative pain was significantly (*p* < 0.00001) more common when only the recumbent approach (17.3%) was used than when only the standing (10.8%) or both approaches were used (1.38%). Further postoperative pain was significantly (*p* < 0.00001) more common when other apparata different than gastrointestinal and urogenital were involved in the procedure (14%) than gastrointestinal (11.6%), urogenital (7.3%) or both (0.8%). Incidence of postoperative pain was not different when CO_2_ insufflation was induced or not (11.8% with CO_2_ insufflation, 12.7% no CO_2_ insufflation, *p* = 0.700).

## 4. Discussion

### 4.1. Summary of the Evidence 

This scoping review reported data from the current available literature on intra and postoperative complications related to laparoscopy and laparoscopic assisted procedures in equids between 1992 and 2022. These data show a paucity of studies reporting the definitions of complications. Furthermore, there is a lack of homogeneity in the criteria used to define each complication in a standardized and reproducible manner, limiting comparison of complication rates between studies and centers over time. Incisional complications and postoperative pain are the most common complications of these procedures. The quality of evidence in equine laparoscopy is low, with most studies being retrospective in nature and having a low caseload.

### 4.2. Reporting of Complications

A high number of intra and postoperative complications have been reported in laparoscopy and laparoscopic-assisted procedure in equids. However, the limited number of randomized studies carries a greater risk of bias in the included studies, and this makes it difficult to define the true morbidity of a surgical procedure [4,8,9].

### 4.3. Definitions of Complications

We found a lack of definitions of both intra- and postoperative complications, and when definitions of complications were found often differed between studies, making difficult to compare them. Adopting rigorous criteria to define and classify complications is fundamental to evaluate the consequences of a specific surgical procedure to make risk-weighted choices and clearly communicate with owners and referring vets. Defining complications related to surgery could have a great influence on conclusions about the safety and harmfulness of the treatment considered, avoiding misunderstandings related to conflicting definitions. A standardization of the definitions also makes it possible to easily compare studies and therefore to be able to more reliably assess the complications associated with a surgical procedure and identification and correction of technical errors.

### 4.4. Classification of Complications

Standardized criteria for defining and grading surgical complications have been proposed in both human and veterinary medicine [7,8,11,13,14,15,16,17,18]. Despite the usefulness of these guidelines, the overall analysis of research studies has revealed a limited knowledge or lack of their application in studies on surgical complications after laparoscopy or laparoscopic-assisted procedure in equids. This review also identified a limited differentiation between intraoperative complications and technical error or difficulty in carrying out a procedure, due to the difficult use of laparoscopic instruments. Only one study reported the term “technical error” related to a problem that occurred during surgery [23], and only two articles reported the term “sequelae” rather than postoperative complication in reference to conditions that occurred as a direct consequence of laparoscopic surgery, such as oedema, skin inflammation and subcutaneous emphysema at the portal [24,25]. It is important to differentiate these events in order to adopt preventive measure and correct treatment. It is essential to recognize which complications are really associated with surgery and which ones are problems that arise unrelated to the procedure performed. In two studies, for example, the onset of neurological signs and myositis were reported as postoperative complications. In both cases, however, they were conditions not associated with the surgery performed but, more likely, with general anesthesia or with the Trendelemburg position used during some of these operations.

### 4.5. Follow-Up

Although most studies (156/164, 95.1%) reported follow-up, only 38/164 (23.1%) studies reported in detail difference between short and long-term follow-up. Furthermore, there was no standard time to report the follow-up period but there were several time ranges. Evaluating postoperative complications in different time frames makes comparisons between studies difficult. Short-term follow up was considered as the period between surgery and discharge from the hospital in most studies (34/38, 89.5%). Reporting outcomes at discharge from the hospital is a good measure of the success of a procedure. However, reporting only short-term complications at the time of discharge or soon after discharge carries a risk of underestimating the true morbidity, reporting an incomplete picture of the complications potentially related to the surgical procedure. Conditions such as portal oedema or emphysema, in fact, frequently occurred immediately after surgery while other problems, such as incisional infection or recurrence of problem, can occur after a longer time. Reporting a standard definition and implementing standardized time frames for follow up will improve the interpretation of outcome measurements and their communication.

### 4.6. Incidence of Complications 

The most reported intraoperative complications were hemorrhage from ovary or mesovary followed by accidental splenic injury. The increased use of vessel sealing devices in recent years should have reduced this complication. Interestingly, percentages of hemorrhage from mesovarium did not differ between various methods of hemostasis (vessel sealing devices, ligatures and surgical staplers) or between different time periods. Prospective, randomized clinical trials would be useful to determine which method of hemostasis would be more indicated in equine laparoscopic ovariectomy.

As with other surgical procedures, incisional complications and postoperative pain are the most frequent postoperative complications. In our study it appears that standing procedures on the urogenital apparatus are less associated with complications than procedures on the gastrointestinal apparatus performed in recumbency. Prospective, randomized clinical trials on methods to reduce incisional infection are warranted in equine surgery.

### 4.7. Limitations

This scoping review has several limitations. An accurate search of the studies was performed by inserting keywords in the databases considered, outlined in the a priori protocol. However, it is possible that some studies may have been excluded due to selection bias, if the title or abstract did not contain a relevant search term, or due to lack of access to the full texts. Further, it has previously been shown that medical databases do not necessarily identify all relevant veterinary publications [15]. Furthermore, full texts in other language than English were not considered and consequently there was a risk of excluding important data. Limitations in the data reported in some articles and potential errors in their evaluation during data abstraction cannot be excluded. Further, only a few studies considered, such as randomized clinical trials or prospective clinical studies (16/164, 9.8%), had high levels of evidence-based medicine. The time interval considered could also represent a limit. In our study, the time period 1992–2022 was chosen because we aimed to evaluate whether classification of complications used in human surgery had been applied by researchers in laparoscopic procedures in equids, since first classifications of complications in human surgery were published in 1992 [3]. However, other time intervals may have led to the different results.

## 5. Conclusions

There is a lack in the adoption of standardized and rigorous schemes to define and classify surgical complications in laparoscopy and laparoscopic-assisted procedures in equids. Moreover, an accurate distinction between complications and other events such as technical errors, sequelae, or cause of complications or complications not related to surgery is needed. There is a need for a classification system in equine surgery. Following a proposal of standardized criteria for reporting and grading complications, an independent and multidisciplinary expert group should make recommendations for reporting complications in equine surgery using rigorous Delphi methodology [26]. Prospective randomized clinical trials are warranted to define methods for reducing intra- and postoperative complications.

## Figures and Tables

**Table 1:** Intraoperative complications reported in the included articles, listed by number of articles, as resulting from a scoping review of the literature on surgical complications related to laparoscopy in equids.

Intraoperative Complications	No. of Articles	% on Total Articles
Hemorrhage from ovary/mesovary	21	12.73
Accidental splenic injury	8	4.85
Parietal hemorrhage	7	4.24
Ovary/testis dropping	6	3.64
Signs of discomfort/pain	5	3.03
Poor visual field	4	2.42
Accidental intestinal injury	3	1.82
Accidental thorax penetration	3	1.82
Need for laparotomy	2	1.21
Accidental uterine injury	2	1.21
Poor portal placement	1	0.61
Suture breakage	1	0.61
Failure of instrument use	1	0.61
Adhesions with pathologic ovary	1	0.61
Ovary burst with abdominal contamination	1	0.61
Respiratory acidosis	1	0.61
Peritoneal detachment	1	0.61
Horse going down in the stocks	1	0.61
Bradycardia/ventricular premature contraction	1	0.61
Failure of intestinal reposition	1	0.61
Rupture of the flap	1	0.61

**Table 2:** Relative and cumulative incidence of intraoperative complications reported in 164 articles as resulting from a scoping review of the literature on surgical complications related to laparoscopy in equids.

Intraoperative Complications	Total N of Cases in All Articles Reporting the Given Complication	N of Cases with the Given Complication	Relative Incidence	Cumulative Incidence (on 2321 Cases)
Hemorrhage from ovary/mesovary	336	56	16.67	2.41
Ovary/testis dropping	130	12	9.23	0.51
Poor visual field	91	11	12.09	0.47
Accidental splenic injury	247	11	4.45	0.47
Signs of discomfort/pain	63	9	14.29	0.38
Peritoneal detachment	40	6	15.00	0.25
Parietal hemorrhage	166	6	3.61	0.25
Accidental intestinal injury	121	4	3.31	0.17
Need for laparotomy	48	4	8.33	0.17
Accidental thorax penetration	25	3	12.00	0.12
Poor portal placement	60	3	5.00	0.12
Respiratory acidosis	2	2	100.00	0.08
Bradycardia/ventricular premature contraction	8	2	25.00	0.08
Suture breakage	12	2	16.67	0.08
Adhesions with pathologic ovary	43	2	4.65	0.08
Accidental uterine injury	85	2	2.35	0.08
Ovary burst with abdominal contamination	43	1	2.33	0.04
Horse going down in the stocks	65	1	1.54	0.04
Failure of intestinal reposition	12	1	8.33	0.04
Rupture of the flap	30	1	3.33	0.04
Failure of instrument use	10	1	10.00	0.04

**Table 3:** Postoperative complications reported in the included articles, listed by number of articles, as resulting from a scoping review of the literature on surgical complications related to laparoscopy in equids.

Postoperative Complications	No. of Articles	% on Total Articles
Incisional complications	106	64.24
Postoperative pain	54	32.73
Fever	25	15.15
Cardiac problems (including tachycardia, AV block, cardiac failure)	11	6.67
Anorexia	7	4.24
Depression	6	3.64
Diarrhea	6	3.64
Declined PCV	5	3.03
Respiratory problems	5	3.03
Adhesions	4	2.42
Bleeding from the portal	3	1.82
Decreased passage of feces	3	1.82
Hemoabdomen	3	1.82
Failure of the procedure	2	1.21
Piroplasmosis	2	1.21
Phlebitis	2	1.21
Recurrence of problem	2	1.21
Vaginal discharge	2	1.21
Hematoma (at gonadectomy site)	2	1.21
Peritonitis	2	1.21
Deterioration of clinical condition	1	0.61
Postoperative dehydration	1	0.61
Esophageal obstruction	1	0.61
Colitis	1	0.61
AST/AMY alteration	1	0.61
Hearth murmur	1	0.61
Azotemia	1	0.61
Dysuria	1	0.61
Rectal tear	1	0.61
Abscess at portal site	1	0.61
Neutrophilia	1	0.61
Pigmenturia	1	0.61
Pleuropneumonia	1	0.61
Lameness	1	0.61

**Table 4:** Relative and cumulative incidence of postoperative complications reported in 164 articles as resulting from a scoping review of the literature on surgical complications related to laparoscopy in equids.

Postoperative Complications	Total N of Cases in All Articles Reporting the Given Complication	N of Cases with the Given Complication	Relative Incidence	Cumulative Incidence (on 2321 Cases)
Incisional complications	2156	477	22.12	20.55
Postoperative pain	1561	131	8.39	5.64
Fever	819	49	5.98	2.11
Decreased passage of feces	31	24	77.42	1.03
Cardiac problems (including tachycardia, AV block, cardiac failure)	90	16	17.78	0.68
Failure of the procedure	269	15	5.58	0.64
Depression	186	13	6.99	0.56
Hemorrhage	366	13	3.55	0.56
Anorexia	70	11	15.71	0.47
Diarrhea	86	10	11.63	0.43
AST/AMY alteration	6	6	100.00	0.25
Declined PCV	12	6	50.00	0.25
Vaginal discharge	11	5	45.45	0.21
Adhesions	196	5	2.55	0.21
Bleeding from the portal	256	4	1.56	0.17
Respiratory problems	3	3	100.00	0.12
Piroplasmosis	18	3	16.67	0.12
Phlebitis	44	3	6.82	0.12
Hematoma (at gonadectomy site)	165	3	1.82	0.12
Recurrence of problem	237	2	0.84	0.08
Peritonitis	244	2	0.82	0.08
Deterioration of clinical condition	1	1	100.00	0.04
Dysuria	1	1	100.00	0.04
Abscess at portal site	1	1	100.00	0.04
Neutrophilia	1	1	100.00	0.04
Pigmenturia	1	1	100.00	0.04
Heart murmur	1	1	100.00	0.04
Esophageal obstruction	6	1	16.67	0.04
Pleuropneumonia	8	1	12.50	0.04
Azotemia	20	1	5.00	0.04
Lameness	32	1	3.13	0.04
Rectal tear	55	1	1.82	0.04
Postoperative dehydration	157	1	0.64	0.04
Colitis	236	1	0.42	0.04

## Data Availability

The authors confirm that the data supporting the findings of this study are available within the article and its Supplementary Materials.

## Supplementary Materials

The following supporting information can be downloaded at: https://www.mdpi.com/article/10.3390/vetsci9100577/s1, Supplementary Material 1: Complete search strings for each database used in a scoping review of intra and postoperative complications related to laparoscopy in equids. Supplementary Material 2: Complete data charting of all sources intra and postoperative complications related to laparoscopy in equids. including a list of 164 references. Supplementary Material 3: Table S1: Definition for signs of pain or discomfort reported in 1 out of 5 articles which reported signs of pain or discomfort as an intraoperative complication. Table S2: Definition for poor portal placement reported in 1 out of 2 articles which reported poor portal placement as an intraoperative complication. Table S3: Definition for failure of the first procedure reported in 1 article out of 2 articles which reported failure of the first procedure as a postoperative complication. Table S4: Definition for anorexia reported in 1 out of 7 articles which reported anorexia as a postoperative complication. Table S5: Definition for postoperative pain reported in 11 out of 54 articles which reported postoperative pain as a postoperative complication. Table S6: Definition for incisional complications in 8 out of 106 articles which reported incisional complications as a postoperative complication. Table S7: Definition for decrease in fecal output in 1 out of 3 articles which reported decrease in fecal output as a postoperative complication. Table S8: Definition for hemoabdomen in 1 out of 3 articles which reported hemoabdomen as a postoperative complication. Table S9: Definition for AST/AMY alteration in 1 article which reported AST/AMY alteration as a postoperative complication. Table S10: Definition for piroplasmosis in 2 out of 2 articles which reported piroplasmosis as a postoperative complication. Table S11: Definition for phlebitis in 1 out of 2 articles which reported phlebitis as a postoperative complication. Table S12: Relative and cumulative incidence of hemorrhage during laparoscopic ovariectomy in horses with vessel sealing devices as hemostasis methods. Table S13: Relative and cumulative incidence of hemorrhage during laparoscopic ovariectomy in horses with other methods of hemostasis (ligatures and surgical staplers).
