# Supplementary material for: Occurrence and Definitions of Intra and Postoperative Complications Related to Laparoscopy in Equids: A Scoping Review
Source: Vet Sci. 2022 Oct 17;9(10):577. doi: 10.3390/vetsci9100577 (PMC9609183; doi:10.3390/vetsci9100577)
Supplement: Supplementary file 1 [file vetsci-09-00577-s001.zip › Supplementary material 1.pdf]

Search strategy

Web of Science: (((ALL=(horse OR equine OR mule OR donkey)) AND ALL=(laparoscopy OR laparoscopies)) AND LA=(English)) AND PY=(1982-2022)

PubMed: (((horse OR equine OR mule OR donkey) AND (laparoscopy OR laparoscopies)) AND (English[Language])) AND (("1982/01/01"[Date - Completion] : "2022/12/31"[Date - Completion]))

CAB: (horse OR equine OR mule OR donkey) AND (laparoscopy OR laparoscopies) AND la:(English) AND yr:[1982 TO 2022]

Scopus: (horse OR equine OR mule OR donkey) AND (laparoscopy OR laparoscopies) AND (LIMIT-TO (LANGUAGE , "English")) AND (EXCLUDE (PUBYEAR , 1981) OR EXCLUDE (PUBYEAR , 1979) OR EXCLUDE (PUBYEAR , 1978) OR EXCLUDE (PUBYEAR , 1977) OR EXCLUDE (PUBYEAR , 1974) OR EXCLUDE (PUBYEAR , 1972) OR EXCLUDE (PUBYEAR , 1970))
