# Supplementary material for: Occurrence and Definitions of Intra and Postoperative Complications Related to Laparoscopy in Equids: A Scoping Review
Source: Vet Sci. 2022 Oct 17;9(10):577. doi: 10.3390/vetsci9100577 (PMC9609183; doi:10.3390/vetsci9100577)
Supplement: Supplementary file 1 [file vetsci-09-00577-s001.zip › Supplementary material 2.pdf]

Article title	**Type of study**	**Location**	**N of cases**	**Apparatus (GI/UG/both /other)**	**Purpose (diagnostic/ treatment/ preventive/experimental)**	**Standing/ Recumbency**	**Pneumoperitoneum**	**Hand assisted**
1. Al-Badrany MS. Laparoscopic ovariectomy in standing donkeys by titanium clips and monopolar electrocautery. *Journal of Animal and Veterinary Advances*. 2007;6(5):663-667.	Experimental in vivo study	Asia	6	UG	Experimental	Standing	x	X
2. Albanese V, Hanson RR, McMaster MA, Koehler JW, Caldwell FJ. Use of a Barbed Knotless Suture for Laparoscopic Ablation of the Nephrosplenic Space in 8 Horses. *Veterinary Surgery*. Aug 2016;45(6):824-830. doi:10.1111/vsu.12520	Experimental in vivo study	North America	8	Other	Preventive	Standing		X
3. Alldredge JG, Hendrickson DA. Use of high-power ultrasonic shears for laparoscopic ovariectomy in mares. Article. *Journal of the American Veterinary Medical Association*. 2004;225(10):1578-1580. doi:10.2460/javma.2004.225.1578	Prospective observational study	North America	10	UG	Treatment	Standing	x	
4. Alsafy MAM, El-Kammar MH, Kassem MM, El-Gendy SAA, El-Khamary AN. Laparoscopic Anatomy of the Abdomen and Laparoscopic Ligating Loops, Electrocoagulation, and a Novel Modified Electroligation Ovariectomy in Standing Mare. *Journal of Equine Veterinary Science*. Nov 2013;33(11):912-923. doi:10.1016/j.jevs.2013.01.007	Experimental in vivo study	Asia	9	UG	Experimental	Standing	x	
5. Arévalo Rodríguez JM, Grulke S, Salciccia A, De La Rebière De Pouyade G. Nephrosplenic space closure significantly decreases recurrent colic in horses: A retrospective analysis. Note. *Veterinary Record*. 2019;185(21):657. doi:10.1136/vr.105458	Retrospective case-control	Europe	42	Other	Preventive	Standing		
6. Aziz DM, Al-Badrany MS, Taha MB. Laparoscopic ovariectomy in standing donkeys by using a new instrument. *Animal Reproduction Science*. Aug 2008;107(1-2):107-114. doi:10.1016/j.anireprosci.2007.06.011	Experimental in vivo study	Asia	6	UG	Experimental	Standing	x	
7. Barrell EA, Kamm JL, Hendrickson DA. Recurrence of renosplenic entrapment after renosplenic space ablation in a seven-year-old stallion. *Journal of the American Veterinary Medical Association*. 2011;239(4):504-507. doi:10.2460/javma.239.4.504	Case report	North America	1	GI	Treatment	Standing	x	
8. Bartmann CP, Lorber KJ. Laparoscopic gonadectomy in two half-sister horses with male pseudohermaphroditism of the testicular feminisation type. *Equine Veterinary Education*. 2003;15(6):299-304. doi:10.1111/j.2042-3292.2003.tb00253.x	Case report	Europe	2	UG	Treatment	Standing	x	X

9. Bleyaert HF, Brown MP, Bonenclark G, Bailey JE. Laparoscopic adhesiolysis in a horse. *Veterinary Surgery*. Nov-Dec 1997;26(6):492-496. doi:10.1111/j.1532-950X.1997.tb00522.x	Case report	North America	1	GI	Treatment	Recumbency	x	X
10. Bont MPd, Wilderjans H, Simon O. Standing laparoscopic ovariectomy technique with intraabdominal dissection for removal of large pathologic ovaries in mares. *Veterinary Surgery*. 2010;39(6):737-741.	Case series	Europe	43	UG	Treatment	Standing	x	X
11. Bouré LP, Pearce SG, Kerr CL, et al. Evaluation of laparoscopic adhesiolysis for the treatment of experimentally induced adhesions in pony foals. Article. *American Journal of Veterinary Research*. 2002;63(2):289-294. doi:10.2460/ajvr.2002.63.289	Experimental in vivo study	North America	8	GI	Experimental	Recumbency	x	X
12. Bouré L, Marcoux M, Laverty S. Laparoscopic abdominal anatomy of foals positioned in dorsal recumbency. Article. *Veterinary Surgery*. 1997;26(1):1-6. doi:10.1111/j.1532-950X.1997.tb01455.x	Experimental in vivo study	North America	2	Other	Experimental	Recumbency	x	X
13. Bouré L, Marcoux M, Laverty S. Paralumbar fossa laparoscopic ovariectomy in horses with use of Endoloop ligatures. Article. *Veterinary Surgery*. 1997;26(6):478-483. doi:10.1111/j.1532-950X.1997.tb00520.x	Experimental in vivo study	North America	7	UG	Treatment	Both	x	
14. Bracamonte JL, Bouré LP, Geor RJ, et al. Evaluation of a laparoscopic technique for collection of serial full-thickness small intestinal biopsy specimens in standing sedated horses. *Am J Vet Res*. Mar 2008;69(3):431-9. doi:10.2460/ajvr.69.3.431	Experimental in vivo study	North America	13	GI	Experimental	Standing	x	X
15. Bracamonte JL, Duke-Novakovski T. A pilot study evaluating laparoscopic closure of the nephrosplenic space using an endoscopic suturing device in standing horses. *Canadian Veterinary Journal-Revue Veterinaire Canadienne*. Jun 2016;57(6):651-654.	Prospective observational study	North America	4	Other	Experimental	Standing	x	
16. Bracamonte JL, Thomas KL. Laparoscopic cryptorchidectomy with a vessel-sealing device in dorsal recumbent horses: 43 cases. Article. *Veterinary Surgery*. 2017;46(4):559-565. doi:10.1111/vsu.12624	Retrospective case series	North America	43	UG	Treatment	Recumbency	x	

17. Brink P, Schumacher J. Elevating the uterus (uteropexy) of five mares by laparoscopically imbricating the mesometrium. *Equine Veterinary Journal*. Nov 2010;42(8):675-679. doi:10.1111/j.2042-3306.2010.00069.x	Retrospective case series	North America	5	UG	Treatment	Standing	x	X
18. Brommer H, Grinwis GCM, van Loon V, Ensink JM. Laparoscopic-assisted diagnosis of anomalous unilateral abdominal cryptorchidism. *Equine Veterinary Education*. Aug 2011;23(8):391-395. doi:10.1111/j.2042-3292.2011.00243.x	Case report	Europe	1	UG	Diagnostic	Standing	x	
19. Brugmans F, Deegen E. Laparoscopic surgical technique for repair of rectal and colonie tears in horses: An experimental study. Article. *Veterinary Surgery*. 2001;30(5):409-416. doi:10.1053/jvet.2001.25864	Experimental in vivo study	Europe	3	GI	Experimental	Recumbency	x	
20. Burke MJ, Parente EJ. Prosthetic Mesh for Obliteration of the Nephrosplenic Space in Horses: 26 Clinical Cases. Article. *Veterinary Surgery*. 2016;45(2):201-207. doi:10.1111/vsu.12434	Retrospective case series	North America	26	Other	Preventive	Standing	x	X
21. Busschers E, Southwood LL, Parente EJ. Laparoscopic diagnosis and correction of a nephrosplenic entrapment of the large colon in a horse. *Equine Veterinary Education*. Mar 2007;19(2):60-63. doi:10.2746/095777307x181889	Case report	North America	1	GI	Diagnostic/treatment	Standing	x	
22. Butt TD, Wilson DG. Laparoscopic colopexy in a horse. *Can Vet J*. Jul 2003;44(7):586-8.	Case report	North America	1	GI	Preventive	Recumbency	x	
23. Carluccio A, de Amicis I, Petrizzi L, et al. Laparoscopic drainage of an ovarian haematoma in a Martina Franca jenny (Equus asinus). Article. *Veterinarni Medicina*. 2020;65(8):358-363. doi:10.17221/29/2020-VETMED	Case report	Europe	1	UG	Treatment	Standing	x	X
24. Caron JP, Brakenhoff J. Intracorporeal suture closure of the internal inguinal and vaginal rings in foals and horses. Article. *Veterinary Surgery*. 2008;37(2):126-131. doi:10.1111/j.1532-950X.2007.00366.x	Case series	North America	8	UG	Treatment/preventive	Recumbency	x	

25. Caron JP, Mehler SJ. Laparoscopic mesh incisional hernioplasty in five horses. Article. *Veterinary Surgery*. 2009;38(3):318-325. doi:10.1111/j.1532-950X.2009.00511.x	Case series	North America	5	Other	Treatment	Recumbency	x	
26. Cokelaere SM, Martens AMJG, Wiemer P. Laparoscopic ovariectomy in mares using a polyamide tie-rap. Article. *Veterinary Surgery*. 2005;34(6):651-656. doi:10.1111/j.1532-950X.2005.00101.x	Prospective observational study	Europe	10	UG	Experimental	Standing	x	X
27. Cokelaere SM, Martens A, Vanschandevijl K, Wilderjans H, Steenhaut M. Hand-assisted laparoscopic nephrectomy after initial ureterocystostomy in a Shire filly with left ureteral ectopia. *Vet Rec*. Sep 22 2007;161(12):424-7. doi:10.1136/vr.161.12.424	Case report	Europe	1	UG	Treatment	Standing		
28. Colbath AC, Hackett ES, Lesser CS, Hendrickson DA. Left paralumbar laparoscopic bilateral ovariectomy in mares. *Veterinary Surgery*. May 2017;46(4):574-579. doi:10.1111/vsu.12637	Prospective observational study	North America	20	UG	Experimental	Standing	x	
29. Collar EM, Duesterdieck-Zellmer KF, Huber MJ, Semevolos SA, Parker JE, Husby KA. Outcome of bilateral equid laparoscopic ovariectomies. Article. *Veterinary Surgery*. 2021;50(5):975-983. doi:10.1111/vsu.13651	Retrospective cohort study	North America	51	UG	Treatment/preventive	Standing	x	X
30. Comino F, Röcken M, Gorvy D. Standing laparoscopy combined with a conventional inguinal approach to treat extended septic funiculitis in 12 horses. *Vet Surg*. Apr 18 2022;doi:10.1111/vsu.13809	Retrospective case series	Multicentric	12	UG	Treatment	Standing	x	
31. Corsalini J, Gialletti R, Lotto E, Nannarone S. Laparoscopic Uteropexy (Mesometrium Imbrication) in Three Mares Using a Barbed Suture. *Journal of Equine Veterinary Science*. May 2016;40:102-105. doi:10.1016/j.jevs.2016.02.236	Prospective observational study	Europe	3	UG	Treatment	Standing	x	
32. Cribb NC, Arroyo LG, Boure L. Standing laparoscopic abdominal lavage using a suction-irrigation device in 2 horses with primary suppurative peritonitis. *Canadian Veterinary Journal-Revue Veterinaire Canadienne*. Apr 2021;62(4):397-402.	Case report	North America	2	Other	Treatment	Standing		

33. Cribb NC, Boure LP. Laparoscopic Removal of a Large Abdominal Testicular Teratoma in a Standing Horse. *Veterinary Surgery*. Jan 2010;39(1):131-135. doi:10.1111/j.1532-950X.2009.00618.x	Case report	North America	1	UG	Treatment	Standing	x	
34. Cribb NC, Koenig J, Sorge U. Comparison of laparoscopic versus conventional open cryptorchidectomies on intraoperative and postoperative complications and duration of surgery, anesthesia, and hospital stay in horses. *Javma-Journal of the American Veterinary Medical Association*. Apr 2015;246(8):885-892. doi:10.2460/javma.246.8.885	Retrospective cohort study	North America	46	UG	Treatment	Standing		
35. Cypher EE, Blackford J, Snowden RT, Sexton JA, Schumacher J. Surgical correction of entrapment of the large colon and caecum through a mesoduodenal rent with standing laparoscopic repair in a mare. *Equine Veterinary Education*. Apr 2020;32(4):185-188. doi:10.1111/eve.12941	Case report	North America	1	GI	Treatment	Standing	x	X
36. Daniel AJ, Easley JT, Story MR, Hendrickson DA, Hackett ES. Standing hand-assisted laparoscopic removal of large granulosa cell tumours in horses using a specimen retrieval bag and morcellator. *Equine Veterinary Education*. Oct 2015;27(10):505-509. doi:10.1111/eve.12374	Case report	North America	3	UG	Treatment	Standing	x	
37. Daniel A, McCue P, Ferris R, Miller C, Leise B. Bilateral ovarian leiomyoma treated by standing laparoscopic ovariectomy. *Equine Veterinary Education*. Oct 2015;27(10):510-514. doi:10.1111/eve.12438	Case report	North America	1	UG	Treatment	Standing	x	
38. Davis EW. Laparoscopic cryptorchidectomy in standing horses. *Veterinary Surgery*. Jul-Aug 1997;26(4):326-331. doi:10.1111/j.1532-950X.1997.tb01505.x	Prospective clinical study	North America	11	UG	Treatment	Standing	x	
39. de Fourmestraux C, Geffroy O, Siliart B, Albaric O, Tessier C. Evaluation of success rate of laparoscopic castration without orchidectomy in 32 mature horses. *Equine Veterinary Education*. Jan 2014;26(1):34-39. doi:10.1111/eve.12089	Retrospective case series	Europe	32	UG	Treatment	Standing		
40. Delcazo M, Geburek F, Kohler K, Rocken M, Theiss F. Laparoscopic resection of an exostosis of the os pubis in a horse. *Veterinary Surgery*. Apr 2020;49(3):614-620. doi:10.1111/vsu.13349	Case report	Europe	1	Other	Treatment	Standing	x	

41. Delli-Rocili MM, Cribb NC, Trout DR, Thomason JJ, Valverde A. Effectiveness of a paraverterbral nerve block versus local portal blocks for laparoscopic closure of the nephrosplenic space: A pilot study. *Veterinary Surgery*. Jul 2020;49(5):1007-1014. doi:10.1111/vsu.13452	Randomized clinical trial	North America	12	Other	Experimental	Standing		
42. Delling U, Howard RD, Pleasant RS, Lanz OI. Hand-assisted laparoscopic ovariohysterectomy in the mare. *Vet Surg*. Sep-Oct 2004;33(5):487-94. doi:10.1111/j.1532-950x.2004.04063.x	Experimental in vivo study	North America	8	UG	Experimental	Recumbency	x	
43. Delling U, Stoebe S, Brehm W. Hand-assisted laparoscopic adhesiolysis of extensive small intestinal adhesions in a mare after breeding injury. *Equine Veterinary Education*. Nov 2012;24(11):545-551. doi:10.1111/j.2042-3292.2011.00333.x	Case report	Europe	1	GI	Treatment	Standing	x	
44. Desmaizières LM, Martinot S, Lepage OM, Bareiss E, Cadoré JL. Complications associated with cannula insertion techniques used for laparoscopy in standing horses. Article. *Veterinary Surgery*. 2003;32(6):501-506. doi:10.1111/j.1532-950X.2003.00501.x	Retrospective cohort study	Europe	40	Other	Diagnostic/treatment	Standing	x	
45. Devick IF, Hendrickson DA. Left paralumbar fossa approach combined with mesocolon fenestration for bilateral equine ovariectomy. *Vet Surg*. Jul 2019;48(5):735-741. doi:10.1111/vsu.13166	Experimental in vivo study	North America	5	UG	Experimental	Standing	x	
46. Devick IF, Leise BS, Rao S, Hendrickson DA. Evaluation of post-operative pain after active desufflation at completion of laparoscopy in mares undergoing ovariectomy. *Canadian Veterinary Journal-Revue Veterinaire Canadienne*. Mar 2018;59(3):261-266.	Prospective observational study	North America	38	UG	Treatment	Standing	x	X
47. Diekstall M, Rijkenhuizen A. Mesorectal hematoma associated with colic and caudal neurological signs in a horse. *Pferdeheilkunde*. May-Jun 2018;34(3):232-236. doi:10.21836/pem20180303	Case report	Europe	1	Other	Diagnostic	Standing	x	
48. Diekstall M, Rohde C, Rijkenhuizen ABM. Post-partum uterine rupture: Standing repair in three mares using a laparoscopic technique. *Equine Veterinary Education*. Jun 2020;32(6):319-324. doi:10.1111/eve.13001	Prospective observational study	Europe	3	UG	Treatment	Standing	x	

49. Düsterdieck KF, Pleasant RS, Lanz OI, Saunders G, Howard RD. Evaluation of the harmonic scalpel for laparoscopic bilateral ovariectomy in standing horses. *Vet Surg*. May-Jun 2003;32(3):242-50. doi:10.1053/jvet.2003.50022	Experimental in vivo study	North America	8	UG	Experimental	Standing	x	X
50. Easley JT, McGilvray KC, Hendrickson DA, Bruemmer J, Hackett ES. Vessel sealer and divider instrument temperature during laparoscopic ovariectomy in horses. Article. *Veterinary Surgery*. 2018;47:O26-O31. doi:10.1111/vsu.12755	Prospective observational study	North America	15	UG	Experimental	Standing	x	X
51. El-Sherif MW, El-Khamary AN, Abdel-Wahed RE. Lateral versus dorsal recumbent laparoscopic cryptorchidectomy and castration in horses. *Online Journal of Veterinary Research*. 2017;21(8):523-530.	Prospective case-control	Asia	20	UG	Treatment	Recumbency		
52. Epstein KL, Parente EJ. Laparoscopic obliteration of the nephrosplenic space using polypropylene mesh in five horses. *Veterinary Surgery*. Jul 2006;35(5):431-437. doi:10.1111/j.1532-950X.2006.00171.x	Experimental in vivo study	North America	5	Other	Experimental	Standing		
53. Farstvedt E, Hendrickson D. Laparoscopic closure of the nephrosplenic space for prevention of recurrent nephrosplenic entrapment of the ascending colon. *Veterinary Surgery*. Nov-Dec 2005;34(6):642-645. doi:10.1111/j.1532-950X.2005.00099.x	Retrospective case series	North America	10	Other	Preventive	Standing	x	
54. Fernández-Parra R, Losada-Floriano A, Zilberstein L, Bourzac C. Iatrogenic pneumothorax-induced heart murmur during standing laparoscopy in a 3-year-old horse. Article. *Equine Veterinary Education*. 2022;doi:10.1111/eve.13641	Case report	Europe	1	Other	Preventive	Standing	x	
55. Finley CJ, Fischer AT. Removal of equine cryptorchid testes through an enlarged umbilical portal in dorsally recumbent horses after intra-abdominal laparoscopic castration. *Equine Veterinary Journal*. doi:10.1111/evj.13483	Retrospective case series	North America	79	UG	Treatment	Recumbency	x	
56. Gablehouse KB, Cary J, Farnsworth K, Ragle CA. Standing laparoscopic-assisted vaginal ovariohysterectomy in a mare. *Equine Veterinary Education*. Jun 2009;21(6):303-306. doi:10.2746/095777309x446603	Case report	North America	1	UG	Treatment	Standing	x	

57. Gandini M, Giusto G, Caramello V, Comino F, Rosso A. Single-port laparoscopic incisional hernia repair in a horse. *Equine Veterinary Education*. Jul 2017;29(7):362-366. doi:10.1111/eve.12547	Case report	Europe	1	Other	Treatment	Recumbency	x	
58. Gandini M, Labate F, Rosso A, Giusto G. Strangulating obstruction of the small intestine by a fibrous band originating on the nephrosplenic ligament. *Equine Veterinary Education*. May 2021;33(5):E146-E148. doi:10.1111/eve.13206	Case report	Europe	1	GI	Treatment	Standing	x	
59. Gandini M, Nannarone S, Giusto G, et al. Laparoscopic nephrosplenic space ablation with barbed suture in eight horses. *Javma-Journal of the American Veterinary Medical Association*. Feb 2017;250(4):431-436. doi:10.2460/javma.250.4.431	Case series	Europe	8	Other	Preventive	Standing		X
60. Gardner AK, Santschi EM, Aeffner F, Pigott JH, Russell DS. Testicular ischaemic necrosis as a cause of equine cryptorchidism. Article. *Equine Veterinary Education*. 2017;29(6):314-317. doi:10.1111/eve.12544	Case report	North America	1	UG	Treatment	Recumbency	x	X
61. Gialletti R, Nannarone S, Gandini M, et al. Comparison of Mesh and Barbed Suture for Laparoscopic Nephrosplenic Space Ablation in Horses. *Animals*. Apr 2021;11(4)1096. doi:10.3390/ani11041096	Retrospective case-control	Europe	28	Other	Preventive	Standing	x	
62. Goodin JT, Rodgerson DH, Gomez JH. Standing Hand-Assisted Laparoscopic Ovariectomy in 65 Mares. Article. *Veterinary Surgery*. 2011;40(1):90-92. doi:10.1111/j.1532-950X.2010.00771.x	Case series	North America	65	UG	Treatment	Standing		
63. Goto A, Tagami M, Kato F, et al. Equine nonneoplastic abnormal ovary in a draft mare with high serum anti-Müllerian hormone: a case study. *Journal of Equine Science*. 2021;32(4):147-151. doi:10.1294/jes.32.147	Case report	Asia	1	UG	Treatment	Standing	x	
64. Gottschalk RD, Berg SSvd. Standing laparoscopically-aided ovariectomy in mares. *Journal of the South African Veterinary Association*. 1997;68(3):102-104.	Case series	Africa	13	UG	Treatment	Standing		

65. Gracia-Calvo LA, Duque J, Balao da Silva C, Ezquerra J, Ortega-Ferrusola C. Testicular perfusion after standing laparoscopic peritoneal flap hernioplasty in stallions. Article. *Theriogenology*. 2015;84(5):797-804. doi:10.1016/j.theriogenology.2015.05.014	Case series	Europe	6	UG	Treatment	Standing		
66. Gracia-Calvo LA, Ezquerra LJ, Martín-Cuervo M, et al. Standing laparoscopic peritoneal flap hernioplasty of the vaginal rings does not modify the sperm production and motility characteristics in intact male horses. *Reproduction in Domestic Animals*. 2014;49(6):1043-1048. doi:10.1111/rda.12434	Case series	Europe	6	UG	Preventive	Standing		
67. Gracia-Calvo LA, Ezquerra LJ, Ortega-Ferrusola C, et al. Histological findings in equine testes one year after standing laparoscopic peritoneal flap ernioplasty. Article. *Veterinary Record*. 2016;178(18):450. doi:10.1136/vr.103236	Case series	Europe	10	Other	Preventive	Standing		
68. Gracia-Calvo L, Martín-Cuervo M, Jiménez J, et al. Development of a technique for standing hand-assisted laparoscopic splenectomy in five horses. Article. *Australian Veterinary Journal*. 2015;93(6):183-188. doi:10.1111/avj.12326	Case series	Europe	5	Other	Experimental	Standing	x	
69. Hand R, Rakestraw P, Taylor T. Evaluation of a vessel-sealing device for use in laparoscopic ovariectomy in mares. *Vet Surg*. May-Jun 2002;31(3):240-4. doi:10.1053/jvet.2002.33482	Prospective clinical study	North America	13	UG	Preventive	Standing	x	
70. Hanrath M, Rodgerson DH. Laparoscopic cryptorchidectomy using electrosurgical instrumentation in standing horses. Article. *Veterinary Surgery*. 2002;31(2):117-124. doi:10.1053/jvet.31049	Retrospective clinical study	North America	10	UG	Treatment	Standing	x	
71. Hanson CA, Galuppo LD. Bilateral laparoscopic ovariectomy in standing mares: 22 Cases. Article. *Veterinary Surgery*. 1999;28(2):106-112. doi:10.1053/jvet.1999.0106	Prospective clinical study	North America	22	UG	Treatment	Standing	x	
72. Hendrickson DA, Wilson DG. Laparoscopic cryptorchid castration in standing horses. *Veterinary Surgery*. Jul-Aug 1997;26(4):335-339. doi:10.1111/j.1532-950X.1997.tb01507.x	Prospective clinical study	North America	8	UG	Diagnostic/Treatment	Standing	x	

73. Hilton HG, Aleman M, Maher O, Peterson TS, Whitcomb MB, Galuppo LD. Hand-assisted laparoscopic nephrectomy in a standing horse for the management of renal cell carcinoma. *Equine Veterinary Education*. May 2008;20(5):239-244. doi:10.2746/095777308x295783	Case report	North America	1	Other	Treatment	Standing	x	X
74. Holmes JM, Nath LC, Muurlink MA. Laparoscopic cauterisation of the testicular arteries to manage haemoperitoneum in a gelding. *Equine Veterinary Education*. Jun 2013;25(6):297-300. doi:10.1111/j.2042-3292.2012.00416.x	Case report	Australia	1	UG	Treatment	Standing		X
75. Hoogmoed LMv, Galuppo LD. Laparoscopic ovariectomy using the Endo-GIA stapling device and Endo-Catch pouches and evaluation of analgesic efficacy of epidural morphine sulfate in 10 mares. *Veterinary Surgery*. 2005;34(6):646-650. doi:10.1111/j.1532-950X.2005.00100.x	Randomized clinical trial	North America	10	UG	Experimental	Standing	x	X
76. Hubert JD, Burba DJ, Moore RM. Evaluation of a vessel-sealing device for laparoscopic granulosa cell tumor removal in standing mares. *Vet Surg*. Jun 2006;35(4):324-9. doi:10.1111/j.1532-950X.2006.00151.x	Retrospective case series	North America	8	UG	Treatment	Standing	x	X
77. Jones ARE, Ragle CA, Anderson D, Scott C. Laparoscopic evaluation of the small intestine in the standing horse: Technique and effects*. Article. *Veterinary Surgery*. 2017;46(6):812-820. doi:10.1111/vsu.12664	Prospective pilot study	North America	5	GI	Experimental	Standing	x	X
78. Jones ARE, Ragle CA, Huggons NA, Tibary AA. Bilateral ovariectomy as a treatment for chronic pyometra in four horses. *Equine Veterinary Education*. Oct 2020;32(10):E189-E193. doi:10.1111/eve.13167	Case series	North America	4	UG	Treatment	Standing		
79. Kadic DTN, Bonilla AG. A two-step ovariohysterectomy with unilateral left flank laparoscopic assistance in a Quarter Horse mare. *Equine Veterinary Education*. Oct 2020;32(10):E199-E202. doi:10.1111/eve.13131	Case report	North America	1	UG	Treatment	Standing	x	
80. Kambayashi Y, Tsuzuki N, Seo J, et al. Evaluation of single-incision laparoscopic ovariectomy in standing mares. *Journal of Equine Veterinary Science*. 2014;34(3):446-450. doi:10.1016/j.jevs.2013.07.006	Case series	Asia	5	UG	Treatment	Standing	x	

81. Kamus L.P. Ruzickova,P. Piat,P. Trencart,A. BonillaUse of barbed suture for thoracoscopic repair of diaphragmatic hernias: Three cases. Equine Vet edu. 2022	Case series	Europe	4	Other	Treatment	Standing		X
82. Keoughan CG, Rodgerson DH, Brown MP. Hand-assisted laparoscopic left nephrectomy in standing horses. *Vet Surg*. May-Jun 2003;32(3):206-12. doi:10.1053/jvet.2003.50028	Prospective clinical study	North America	8	UG	Treatment	Standing		
83. Klohnen A, Wilson DG. Laparoscopic repair of scrotal hernia in two foals. *Vet Surg*. Sep-Oct 1996;25(5):414-16. doi:10.1111/j.1532-950x.1996.tb01437.x	Case report	North America	2	GI	Treatment	Recumbency	x	
84. Koch DW, Easley JT, Hatzel JN, et al. Prospective randomized investigation of topical anesthesia during unilateral laparoscopic ovariectomy in horses. *Vet Surg*. Jun 2020;49 Suppl 1:O54-o59. doi:10.1111/vsu.13264	Randomized clinical trial	North America	15	UG	Experimental	Standing	x	
85. Köllmann M, Rötting A, Heberling A, Sieme H. Laparoscopic techniques for investigating the equine oviduct. *Equine Vet J*. Jan 2011;43(1):106-11. doi:10.1111/j.2042-3306.2010.00143.x	Prospective clinical study	Europe	10	UG	Experimental	Standing	x	
86. Kummer M, Theiss F, Jackson M, Fürst A. Evaluation of a Motorized Morcellator for Laparoscopic Removal of Granulosa-Theca Cell Tumors in Standing Mares. Article. *Veterinary Surgery*. 2010;39(5):649-653. doi:10.1111/j.1532-950X.2010.00688.x	Case series	Europe	7	UG	Treatment	Standing	x	
87. Lacitignola L, Imperante A, De Siena R, et al. Wound Retractor Laparoscopic Port System for Standing Laparoscopic Cryptorchidectomy in the Horse: A Case Report. *Journal of Equine Veterinary Science*. Sep 2020;92103168. doi:10.1016/j.jevs.2020.103168	Case report	Europe	1	UG	Treatment	Standing	x	X
88. Lawless SP, Moorman VJ, Hendrickson DA, Mama KR. Comparison of sedation quality and safety of detomidine and romifidine as a continuous rate infusion for standing elective laparoscopic ovariectomy in mares. *Vet Surg*. Jul 2021;50(5):990-998. doi:10.1111/vsu.13627	Prospective clinical study	North America	18	UG	Experimental	Standing		

89. Lloyd D, Walmsley JP, Greet TRC, Payne RJ, Newton JR, Phillips TJ. Electrosurgery as the sole means of haemostasis during the laparoscopic removal of pathologically enlarged ovaries in mares: a report of 55 cases. *Equine Veterinary Journal*. May 2007;39(3):210-214. doi:10.2746/042516407x17116522	Case series	Europe	55	UG	Treatment	Standing		
90. Lund CM, Ragle CA, Dylan Lutter J, Farnsworth KD. Use of a motorized morcellator for elective bilateral laparoscopic ovariectomy in standing equids: 30 cases (2007-2013). Article. *Journal of the American Veterinary Medical Association*. 2014;244(10):1191-1197. doi:10.2460/javma.244.10.1191	Retrospective case series	North America	30	UG	Treatment	Standing		
91. Lund CM, Ragle CA, Lutter JD. Laparoscopic removal of a bladder urolith in a standing horse. *J Am Vet Med Assoc*. Nov 1 2013;243(9):1323-8. doi:10.2460/javma.243.9.1323	Case report	North America	1	UG	Treatment	Standing		
92. Mariën T. Standing laparoscopic herniorrhaphy in stallions using cylindrical polypropylene mesh prosthesis. *Equine Vet J*. Jan 2001;33(1):91-6. doi:10.2746/042516401776767476	Case series	Europe	9	GI	Treatment	Standing	x	
93. Mariën T, Adriaenssen A, Hoeck FV, Segers L. Laparoscopic closure of the renosplenic space in standing horses. Article. *Veterinary Surgery*. 2001;30(6):559-563. doi:10.1053/jvet.2001.28436	Prospective clinical study	Europe	5	Other	Experimental	Standing	x	
94. Martin-Flores M, Campoy L, Kinsley MA, Mohammed HO, Gleed RD, Cheetham J. Analgesic and gastrointestinal effects of epidural morphine in horses after laparoscopic cryptorchidectomy under general anesthesia. Article. *Veterinary Anaesthesia and Analgesia*. 2014;41(4):430-437. doi:10.1111/vaa.12133	Randomized clinical trial	North America	18	UG	Treatment	Recumbency		
95. McNally TP, Rodgerson DH, Lu KG. Infertility in a mare with a chronic uterine tear, diagnosis and successful standing hand-assisted laparoscopic repair. *Equine Veterinary Education*. Sep 2012;24(9):439-443. doi:10.1111/j.2042-3292.2011.00308.x	Case report	North America	1	UG	Treatment	Standing		
96. Mehl ML, Ragle CA. Retroflexion of the large colon in a stallion. *Equine Veterinary Education*. 2001;13(3):116-118. doi:10.1111/j.2042-3292.2001.tb00073.x	Case report	North America	1	GI	Treatment	Standing	x	X

97. Merchan A, Barrett EJ, Rodgerson DH. Hand-assisted laparoscopic cystotomy for cystic calculus removal in male horses (3 cases). *Canadian Veterinary Journal-Revue Veterinaire Canadienne*. Jan 2021;62(1):22-26.	Retrospective case series	Multicentric	3	UG	Treatment	Recumbency	x	
98. Muňoz J, Bussy C. Standing hand-assisted laparoscopic treatment of left dorsal displacement of the large colon and closure of the nephrosplenic space. Article. *Veterinary Surgery*. 2013;42(5):595-599. doi:10.1111/j.1532-950X.2012.01050.x	Case series	Europe	12	Other	Treatment	Standing		X
99. Munsterman AS, Hanson RR, Cattley RC, Barrett EJ, Albanese V. Surgical Technique and Short-Term Outcome for Experimental Laparoscopic Closure of the Epiploic Foramen in 6 Horses. *Veterinary Surgery*. Feb 2014;43(2):105-113. doi:10.1111/j.1532-950X.2013.12116.x	Experimental in vivo study	North America	6	GI	Experimental	Standing	x	X
100. Muurlink T, Walmsley J, Whitton C. Successful laparoscopic surgery for a uterine leiomyoma in a mare. *Equine Veterinary Education*. Oct 2008;20(10):508-511. doi:10.2746/095777308x345642	Case report	Europe	1	UG	Treatment	Standing	x	
101. Nelson BB, Ruple-Czerniak AA, Hendrickson DA, Hackett ES. Laparoscopic Closure of the Nephrosplenic Space in Horses with Nephrosplenic Colonic Entrapment: Factors Associated with Survival and Colic Recurrence. Article. *Veterinary Surgery*. 2016;45:O60-O69. doi:10.1111/vsu.12549	Retrospective cohort study	North America	49	GI	Treatment	Standing	x	X
102. O'Neill HD. Hand-assisted laparoscopic reattachment of a mesoduodenojejunal defect in a broodmare. *Equine Veterinary Education*. 2019;32(10):e184-e188. doi:10.1111/eve.13110	Case report	Europe	1	GI	Treatment	Standing	x	X
103. O'Neill HD, Ricardi G. Concurrent repair of a displaced rib fracture and splenic capsular tearing using laparoscopic technique in a standing horse with acute haemoperitoneum. Article. *Equine Veterinary Education*. 2020;32(4):O19-O23. doi:10.1111/eve.13021	Case report	Europe	1	Other	Treatment	Standing	x	X
104. Ortis HA, Foss RR, McCue PM, Bradecamp EA, Ferris RA, Hendrickson DA. Laparoscopic application of PGE2 to the uterine tube surface enhances fertility in selected subfertile mares. *Journal of Equine Veterinary Science*. 2013;33(11):896-900. doi:10.1016/j.jevs.2013.01.003	Prospective observational study	North America	28	UG	Treatment	Standing	x	

105. Ortved KF, Witte S, Fleming K, Nash J, Woolums AR, Peroni JF. Laparoscopic-assisted splenectomy in a horse with splnomegaly. *Equine Veterinary Education*. Jul 2008;20(7):357-361. doi:10.2746/095777308x320270	Case report	North America	1	Other	Treatment	Recumbency	x	
106. Pader K, Freeman LJ, Constable PD, Wu CC, Snyder PW, Lescun TB. Comparison of Transvaginal Natural Orifice Transluminal Endoscopic Surgery (NOTES) and Laparoscopy for Elective Bilateral Ovariectomy in Standing Mares. Article. *Veterinary Surgery*. 2011;40(8):998-1008. doi:10.1111/j.1532-950X.2011.00877.x	Experimental in vivo study	North America	6	UG	Experimental	Standing	x	X
107. Pepe M, Gialletti R, Moriconi F, Puccetti M, Nannarone S, Singer ER. Laparoscopic sterilization of Sardinia donkeys using an endoscopic stapler. *Vet Surg*. May-Jun 2005;34(3):260-4. doi:10.1111/j.1532-950x.2005.00039.x	Prospective clinical study	Europe	6	UG	Treatment	Both	x	X
108. Petrizzi L, Guerri G, Stratico P, et al. Laparoscopic Ovariectomy in Standing Mule Mares. *Journal of Equine Veterinary Science*. Jan 2020;84102857. doi:10.1016/j.jevs.2019.102857	Prospective observational study	Europe	10	UG	Treatment	Standing	x	
109. Pye J, Galuppo L, Whitcomb MB, Clothier K, Byrne B. Isolation of campylobacter fetus subspecies fetus from an abdominal abscess in an adult mare. Article. *Canadian Veterinary Journal*. 2020;61(12):1307-1311.	Case report	North America	1	Other	Diagnostic/Treatment	Standing		X
110. Quere E, Bourzac C, Farfan M, Losada A, Volmer C, Mespoulhes-Riviere C. Standing Hand-Assisted Laparoscopic Diagnosis and Treatment of a Rare Case of Uterine Adenocarcinoma in an 18-Year-Old Mare. *Journal of Equine Veterinary Science*. Aug 2019;79:39-44. doi:10.1016/j.jevs.2019.05.021	Case report	Europe	1	UG	Treatment	Standing	x	X
111. Radcliffe RM, Manchester AC, Mohammed HO, et al. Laparoscopic-Guided Compared to Skilled Instructor Support for Student Rectal Examination Training Using Live Horses in the Veterinary Curriculum. *Veterinary Surgery*. Apr 2015;44(3):352-358. doi:10.1111/j.1532-950X.2014.12278.x	Randomized clinical trial	North America	Not specified	Other	Experimental/ teaching	Standing	x	
112. Ragle CA, Schneider RK. Ventral Abdominal Approach for Laparoscopic Ovariectomy in Horses. Article. *Veterinary Surgery*. 1995;24(6):492-497. doi:10.1111/j.1532-950X.1995.tb01360.x	Prospective observational study	North America	15	UG	Treatment	Recumbency	x	X

113. Ragle CA, Yiannikouris S, Tibary AA, Fransson BA. Use of a barbed suture for laparoscopic closure of the internal inguinal rings in a horse. *Javma-Journal of the American Veterinary Medical Association*. Jan 2013;242(2):249-253. doi:10.2460/javma.242.2.249	Case report	North America	1	GI	Treatment	Standing	x	X
114. Rambags BPB, Stout TAE, Rijkenhuizen ABM. Ovarian granulosa cell tumours adherent to other abdominal organs; surgical removal from 2 Warmblood mares. *Equine Veterinary Journal*. Sep 2003;35(6):627-632. doi:10.2746/042516403775467261	Case report	Europe	2	UG	Treatment	Standing	x	X
115. Rijkenhuizen ABM. Laparoscopic repair of a traumatic ventral abdominal hernia using a mesh. *Equine Veterinary Education*. 2005;17(5):243-247. doi:10.1111/j.2042-3292.2005.tb00383.x	Case report	Europe	1	Other	Treatment	Recumbency	x	
116. Rijkenhuizen ABM, Dijk Pv. Diagnostic and therapeutic laparoscopy in the horse: experiences in 236 cases. *Pferdeheilkunde*. 2002;18(1):12-20.	Retrospective cohort study	Europe	236	Both	Diagnostic/Treatment	Both		
117. Rijkenhuizen ABM, Goehring L, Lankveld DPK. Laparoscopic repair of a bladder rupture in 2 foals. *Pferdeheilkunde*. Jan-Feb 2003;19(1):9-15. doi:10.21836/pem20030101	Case report	Europe	2	UG	Treatment	Recumbency	x	X
118. Rijkenhuizen ABM, Lichtenberg D, Weitkamp K. Cystic intra-abdominal testicles: Standing laparoscopic removal in two colts. *Equine Veterinary Education*. Aug 2020;32(8):E130-E135. doi:10.1111/eve.13030	Case report	Europe	2	UG	Treatment	Standing	x	X
119. Rijkenhuizen ABM, Loon TJAMv, Boswinkel M. Laparoscopic repair of a ruptured bladder in an adult mare. *Equine Veterinary Education*. 2008;20(4):183-189. doi:10.2746/095777308X292137	Case report	Europe	1	UG	Treatment	Recumbency	x	X
120. Rijkenhuizen ABM, van der Harst MR. Castration in the standing horse combining laparoscopic and conventional techniques. *Equine Veterinary Journal*. Nov 2017;49(6):776-779. doi:10.1111/evj.12681	Case series	Europe	15	UG	Treatment	Standing	x	

121. Rocken M, Mosel G, Barske K, Witte TS. Thoracoscopic Diaphragmatic Hernia Repair in a Warmblood Mare. *Veterinary Surgery*. Jun 2013;42(5):591-594. doi:10.1111/j.1532-950X.2013.12009.x	Case report	Europe	1	Other	Treatment	Standing		X
122. Röcken M, Mosel G, Seyrek-Intas K, et al. Unilateral and bilateral laparoscopic ovariectomy in 157 mares: a retrospective multicenter study. *Vet Surg*. Dec 2011;40(8):1009-14. doi:10.1111/j.1532-950X.2011.00884.x	Retrospective case series	Multicentric	157	UG	Treatment	Standing		
123. Röcken M, Mosel G, Stehle C, Rass J, Litzke LF. Left- and right-sided laparoscopic-assisted nephrectomy in standing horses with unilateral renal disease. *Vet Surg*. Aug 2007;36(6):568-72. doi:10.1111/j.1532-950X.2007.00306.x	Case report	Europe	3	UG	Treatment	Standing	x	
124. Röcken M, Schubert C, Mosel G, Litzke LF. Indications, surgical technique, and long-term experience with laparoscopic closure of the nephrosplenic space in standing horses. *Veterinary Surgery*. 2005;34(6):637-641. doi:10.1111/j.1532-950X.2005.00098.x	Retrospective cohort study	Europe	44	Other	Treatment	Standing	x	X
125. Röcken M, Stehle C, Mosel G, Rass J, Litzke LF. Laparoscopic-assisted cystotomy for urolith removal in geldings. *Vet Surg*. Jun 2006;35(4):394-7. doi:10.1111/j.1532-950X.2006.00163.x	Case report	Europe	4	UG	Treatment	Recumbency	x	
126. Rodgerson DH, Belknap JK, Wilson DA. Laparoscopic ovariectomy using sequential electrocoagulation and sharp transection of the equine mesovarium. Article. *Veterinary Surgery*. 2001;30(6):572-579. doi:10.1053/jvet.2001.28435	Prospective observational study	North America	23	UG	Experimental	Standing	x	
127. Rodgerson DH, Brown MH, Watt BC, Keoughan CG, Hanrath M. Hand-assisted laparoscopic technique for removal of ovarian tumors in standing mares. *Journal of the American Veterinary Medical Association*. May 2002;220(10):1503-+. doi:10.2460/javma.2002.220.1503	Case series	North America	17	UG	Treatment	Standing	x	X
128. Rodgerson DH, Hanson RR. Ligature slippage during standing laparoscopic ovariectomy in a mare. *Can Vet J*. May 2000;41(5):395-7.	Case report	North America	1	UG	Treatment	Standing	x	

129. Roessner HA, Kurtz KA, Caron JP. Laparoscopic ovariectomy diminishes estrus-associated behavioral problems in mares. *Journal of Equine Veterinary Science*. 2015;35(3):250-253. doi:10.1016/j.jevs.2015.01.007	Case series	North America	20	UG	Treatment	Standing	x	
130. Romero A, Rodgerson DH, Fontaine GL. Hand-assisted laparoscopic removal of a nephroblastoma in a horse. *Can Vet J*. Jun 2010;51(6):637-9.	Case report	North America	1	UG	Treatment	Standing		
131. Rossignol F, Mespoulhes-Riviere C, Vitte A, Lechartier A, Boening KJ. Standing Laparoscopic Inguinal Hernioplasty Using Cyanoacrylate for Preventing Recurrence of Acquired Strangulated Inguinal Herniation in 10 Stallions. *Veterinary Surgery*. Jan 2014;43(1):6-11. doi:10.1111/j.1532-950X.2013.12083.x	Case series	Europe	10	GI	Treatment	Standing	x	
132. Rossignol F, Perrin R, Boening KJ. Laparoscopic hernioplasty in recumbent horses using transposition of a peritoneal flap. *Veterinary Surgery*. Aug 2007;36(6):557-562. doi:10.1111/j.1532-950X.2007.00282.x	Prospective clinical study	Europe	13	Other	Experimental/ treatment	Recumbency	x	X
133. Rubio-Martinez LM. Standing laparoscopic castration in an equine male pseudohermaphrodite. *Equine Veterinary Education*. Oct 2012;24(10):507-510. doi:10.1111/j.2042-3292.2011.00330.x	Case report	Africa	1	UG	Treatment	Standing	x	X
134. Sassot LN, Ragle CA, Farnsworth KD, Lund CM. Morcellation for testes extraction in horses undergoing standing laparoscopic cryptorchidectomy. Article. *Canadian Veterinary Journal*. 2017;58(11):1215-1220.	Case report	North America	30	UG	Treatment	Standing	x	
135. Sherlock CE, Lott-Ellis K, Bergren A, Withers JM, Fews D, Mair TS. Granulosa cell tumours in the mare: A review of 52 cases. *Equine Veterinary Education*. Feb 2016;28(2):75-82. doi:10.1111/eve.12449	Retrospective case series	Europe	38	UG	Treatment	Standing		
136. Smith LJ, Mair TS. Unilateral and bilateral laparoscopic ovariectomy of mares by electrocautery. *Veterinary Record*. 2008;163(10):297-300.	Case series	Europe	12	UG	Treatment	Standing	x	X

137. Smith LJ, Perkins JD. Laparoscopic-assisted castration of a monorchid male pseudohermaphrodite pony. *Equine Veterinary Education*. Jun 2009;21(6):295-299. doi:10.2746/095777309x422979	Case report	Europe	1	UG	Treatment	Recumbency	x	
138. Spagnolo JD, Castro LM, Correa RR, et al. Nephrosplenic Space Ablation in Horses After Homologous Pericardium Implant Using a Laparoscopic Stapler. *Journal of Equine Veterinary Science*. Dec 2020;95103275. doi:10.1016/j.jevs.2020.103275	Experimental in vivo study	South America	6	Other	Experimental	Standing	x	
139. Spagnolo JD, Sinhorini IL, Baccarin RYA, et al. Internal inguinal ring closure by laparoscopy using homologous pericardium grafts in horses. *Ciência Rural*. 2016;46(2):318-323. doi:10.1590/0103-8478cr20150042	Experimental in vivo study	South America	6	Other	Experimental	Recumbency	x	
140. Spanton JA, Mair YS, Krudewig G. Pancreatic adenocarcinoma in a donkey. Use of laparoscopy to aid the diagnosis. *Equine Veterinary Education*. Jan 2009;21(1):19-24. doi:10.2746/095777308x382641	Case report	Europe	1	GI	Diagnostic	Standing	x	
141. Stewart SG, Johnston JK, Parente EJ. Hand-assisted laparoscopic repair of a grade IV rectal tear in a postparturient mare. *J Am Vet Med Assoc*. Oct 1 2014;245(7):816-20. doi:10.2460/javma.245.7.816	Case report	North America	1	GI	Treatment	Standing	x	
142. Straticò P, Suriano R, Sciarrini C, Varasano V, Petrizzi L. Laparoscopic-assisted cystotomy and cystostomy for treatment of cystic calculus in a gelding. *Vet Surg*. Jul 2012;41(5):634-7. doi:10.1111/j.1532-950X.2011.00946.x	Case report	Europe	1	UG	Treatment	Recumbency	x	
143. Straticò P, Varasano V, Guerri G, Celani G, Palozzo A, Petrizzi L. A retrospective study of cryptorchidectomy in horses: Diagnosis, treatment, outcome and complications in 70 cases. Article. *Animals*. 2020;10(12):1-10. 2446. doi:10.3390/ani10122446	Case series	Europe	44	UG	Treatment	Standing		X
144. Sutter WW, Hardy J. Laparoscopic repair of a small intestinal mesenteric rent in a broodmare. *Veterinary Surgery*. Jan-Feb 2004;33(1):92-95. doi:10.1111/j.1532-950x.2004.04012.x	Case report	North America	6	GI	Treatment	Standing	x	X

145. Tate LP, Jr., Fogle CA, Bailey CS, Tate KB, Davis JW. Laparoscopic-assisted colpotomy for ovariectomy in the mare. *Veterinary Surgery*. 2012;41(5):625-628. doi:10.1111/j.1532-950X.2012.00993.x	Case series	North America	4	UG	Treatment	Standing	x	X
146. Teixeira PPM, Fidelis OL, Medeiros RM, et al. Laparoscopic Adhesiolysis in a Mini Pony. *Acta Scientiae Veterinariae*. 2013;413.	Case report	South America	1	Other	Treatment	Recumbency	x	X
147. Teixeira PPM, Medeiros RM, Silva MAM, et al. Laparoscopic-assisted Approach to the Pelvic Flexure for Surgical Treatment of Sand Impaction in a Horse. *Acta Scientiae Veterinariae*. 2015;4375.	Case report	South America	1	GI	Treatment	Recumbency	x	
148. Teixeira PPM, Silva MAM, Coutinho LN, et al. ADAPT(tm) trocar use for laparoscopic procedures in equidea. *Arquivo Brasileiro de Medicina Veterinária e Zootecnia*. 2016;68(1):243-246. doi:10.1590/1678-4162-7861	Experimental in vivo study	South America	15	other	Experimental	Standing	x	X
149. Trostle SS, White NA, Donaldson L, Freeman LJ, Hendrickson DA. Laparoscopic colopexy in horses. Article. *Veterinary Surgery*. 1998;27(1):56-63. doi:10.1111/j.1532-950X.1998.tb00098.x	Experimental in vivo study	North America	6	GI	Experimental	Recumbency	x	X
150. Trumble TN, Ingle-Fehr J, Hendrickson DA. Laparoscopic intra-abdominal ligation of the testicular artery following castration in a horse. *Journal of the American Veterinary Medical Association*. May 2000;216(10):1596-+. doi:10.2460/javma.2000.216.1596	Case report	North America	1	UG	Treatment	Recumbency	x	X
151. Tuohy JL, Hendrickson DA, Hendrix SM, Bohanon TC. Standing laparoscopic repair of a ruptured urinary bladder in a mature draught horse. *Equine Veterinary Education*. May 2009;21(5):257-261. doi:10.2746/095777309x409884	Case report	North America	1	UG	Treatment	Standing	x	X
152. van Bergen T, Wiemer P, Bosseler L, Ugahary F, Martens A. Development of a new laparoscopic Foramen Epiploicum Mesh Closure (FEMC) technique in 6 horses. *Equine Veterinary Journal*. May 2016;48(3):331-337. doi:10.1111/evj.12427	Experimental in vivo study	Europe	6	GI	Experimental	Standing	x	

153. van Bergen T, Wiemer P, Schauvliege S, Paulussen E, Ugahary F, Martens A. Laparoscopic Evaluation of the Epiploic Foramen after Celiotomy for Epiploic Foramen Entrapment in the Horse. *Veterinary Surgery*. Jul 2016;45(5):596-601. doi:10.1111/vsu.12493	Case series	Europe	7	GI	Treatment	Standing		X
154. Vitoria A, Romero A, Fuente S, Barrachina L, Vazquez FJ. Application of a laparoscopic technique for vasectomy in standing horses. *Veterinary Record*. Sep 2019;185(11)doi:10.1136/vetrec-2019-105396	Experimental in vivo study	Europe	4	UG	Experimental	Standing	x	
155. Vitte A, Mespoulhès-Rivière C, Lechartier A, Rossignol F. Removal of cystic calculi using a transinguinal laparoscopic-assisted technique in two stallions. Article. *Equine Veterinary Education*. 2013;25(11):573-577. doi:10.1111/eve.12013	Case report	Europe	2	UG	Treatment	Recumbency	x	
156. Vitte A, Rossignol F, Mespoulhes-Rivière C, Lechartier A, Röecken M. Two-Step Surgery Combining Standing Laparoscopy With Recumbent Ventral Median Celiotomy for Removal of Enlarged Pathologic Ovaries in 20 Mares. Article. *Veterinary Surgery*. 2014;43(6):663-667. doi:10.1111/j.1532-950X.2014.12229.x	Case series	Europe	20	UG	Treatment	Both	x	
157. Voermans M, Rijkenhuizen ABM, Velden MAvd. The complex blood supply to the equine testis as a cause of failure in laparoscopic castration. *Equine Veterinary Journal*. 2006;38(1):35-39. doi:10.2746/042516406775374234	Case series	Europe	241	UG	Treatment	Standing		X
158. Waguespack R, Belknap J, Williams A. Laparoscopic management of postcastration haemorrhage in a horse. *Equine Vet J*. Sep 2001;33(5):510-3. doi:10.2746/042516401776254772	Case report	North America	1	UG	Treatment	Standing	x	
159. Walesby HA, Ragle CA, Booth LC. Laparoscopic repair of ruptured urinary bladder in a stallion. *J Am Vet Med Assoc*. Dec 15 2002;221(12):1737-41, 1715. doi:10.2460/javma.2002.221.1736	Case report	North America	1	UG	Treatment	Recumbency	x	X
160. Wilderjans H, Meulyzer M. Laparoscopic closure of the vaginal rings in the standing horse using a tacked intraperitoneal slitted mesh (TISM) technique. *Equine Veterinary Journal*. 2021;54(2):359-367. doi:10.1111/evj.13454	Retrospective case series	Europe	17	GI	Preventive	Standing	x	X

161. Wilderjans H, Meulyzer M, Simon O. Standing Laparoscopic Peritoneal Flap Hernioplasty Technique for Preventing Recurrence of Acquired Strangulating Inguinal Herniation in Stallions. Article. *Veterinary Surgery*. 2012;41(2):292-299. doi:10.1111/j.1532-950X.2011.00914.x	Case series	Europe	30	GI	Preventive	Standing	x	
162. Witte TH, Wilke M, Stahl C, Jandová V, Haralambus R, Straub R. Use of a hand-assisted laparoscopic surgical technique for closure of an extensive mesojejunal rent in a horse. *J Am Vet Med Assoc*. Oct 15 2013;243(8):1166-9. doi:10.2460/javma.243.8.1166	Case report	Europe	1	GI	Treatment	Standing	x	
163. Woodford NS, Payne RJ, McCluskie LK. Laparoscopically-assisted ovariohysterectomy in three mares with pyometra. *Equine Veterinary Education*. Feb 2014;26(2):75-78. doi:10.1111/eve.12094	Case report	Europe	3	UG	Treatment	Both	x	
164. Worsman FCF, Barakzai SZ, Bont MPd, Turner S, Rubio-Martínez LM. Treatment of haemoperitoneum secondary to ruptured granulosa cell tumours in two mares. *Equine Veterinary Education*. 2020;32(2):71-77.	Case report	Multicentric	1	UG	Treatment	Both		
165.								X
166.								
167.								
168.								

169.								X
170.								
171.								
172.								X
173.								X
174.								
175.								
176.								X

177.								
178.								
179.								x
180.								
181.								
182. **References**	**Type of study**	**Location**	**N of cases**	**Apparatus (GI/UG/ other)**	**Purpose (diagnostic/ treatment/ preventive/experimental)**	**Standing/ Recumbency**	**Pneumoperitoneum**	
183. Al-Badrany MS. Laparoscopic ovariectomy in standing donkeys by titanium clips and monopolar electrocautery. *Journal of Animal and Veterinary Advances*. 2007;6(5):663-667.	Experimental in vivo study	Asia	6	UG	Experimental	Standing	x	X
184. Albanese V, Hanson RR, McMaster MA, Koehler JW, Caldwell FJ. Use of a Barbed Knotless Suture for Laparoscopic Ablation of the Nephrosplenic Space in 8 Horses. *Veterinary Surgery*. Aug 2016;45(6):824-830. doi:10.1111/vsu.12520	Experimental in vivo study	North America	8	Other	Preventive	Standing		

185. Alldredge JG, Hendrickson DA. Use of high-power ultrasonic shears for laparoscopic ovariectomy in mares. Article. *Journal of the American Veterinary Medical Association*. 2004;225(10):1578-1580. doi:10.2460/javma.2004.225.1578	Prospective observational study	North America	10	UG	Treatment	Standing	x	
186. Alsafy MAM, El-Kammar MH, Kassem MM, El-Gendy SAA, El-Khamary AN. Laparoscopic Anatomy of the Abdomen and Laparoscopic Ligating Loops, Electrocoagulation, and a Novel Modified Electroligation Ovariectomy in Standing Mare. *Journal of Equine Veterinary Science*. Nov 2013;33(11):912-923. doi:10.1016/j.jevs.2013.01.007	Experimental in vivo study	Asia	9	UG	Experimental	Standing	x	
187. Arévalo Rodríguez JM, Grulke S, Salciccia A, De La Rebière De Pouyade G. Nephrosplenic space closure significantly decreases recurrent colic in horses: A retrospective analysis. Note. *Veterinary Record*. 2019;185(21):657. doi:10.1136/vr.105458	Retrospective case-control	Europe	42	Other	Preventive	Standing		
188. Aziz DM, Al-Badrany MS, Taha MB. Laparoscopic ovariectomy in standing donkeys by using a new instrument. *Animal Reproduction Science*. Aug 2008;107(1-2):107-114. doi:10.1016/j.anireprosci.2007.06.011	Experimental in vivo study	Asia	6	UG	Experimental	Standing	x	X
189. Barrell EA, Kamm JL, Hendrickson DA. Recurrence of renosplenic entrapment after renosplenic space ablation in a seven-year-old stallion. *Journal of the American Veterinary Medical Association*. 2011;239(4):504-507. doi:10.2460/javma.239.4.504	Case report	North America	1	GI	Treatment	Standing	x	
190. Bartmann CP, Lorber KJ. Laparoscopic gonadectomy in two half-sister horses with male pseudohermaphroditism of the testicular feminisation type. *Equine Veterinary Education*. 2003;15(6):299-304. doi:10.1111/j.2042-3292.2003.tb00253.x	Case report	Europe	2	UG	Treatment	Standing	x	
191. Bleyaert HF, Brown MP, Bonenclark G, Bailey JE. Laparoscopic adhesiolysis in a horse. *Veterinary Surgery*. Nov-Dec 1997;26(6):492-496. doi:10.1111/j.1532-950X.1997.tb00522.x	Case report	North America	1	GI	Treatment	Recumbency	x	X
192. Bont MPd, Wilderjans H, Simon O. Standing laparoscopic ovariectomy technique with intraabdominal dissection for removal of large pathologic ovaries in mares. *Veterinary Surgery*. 2010;39(6):737-741.	Case series	Europe	43	UG	Treatment	Standing	x	

193. Bouré LP, Pearce SG, Kerr CL, et al. Evaluation of laparoscopic adhesiolysis for the treatment of experimentally induced adhesions in pony foals. Article. *American Journal of Veterinary Research*. 2002;63(2):289-294. doi:10.2460/ajvr.2002.63.289	Experimental in vivo study	North America	8	GI	Experimental	Recumbency	x	X
194. Bouré L, Marcoux M, Laverty S. Laparoscopic abdominal anatomy of foals positioned in dorsal recumbency. Article. *Veterinary Surgery*. 1997;26(1):1-6. doi:10.1111/j.1532-950X.1997.tb01455.x<br>195. Bouré L, Marcoux M, Laverty S. Paralumbar fossa laparoscopic ovariectomy in horses with use of Endoloop ligatures. Article. *Veterinary Surgery*. 1997;26(6):478-483. doi:10.1111/j.1532-950X.1997.tb00520.x<br>196. Bracamonte JL, Bouré LP, Geor RJ, et al. Evaluation of a laparoscopic technique for collection of serial full-thickness small intestinal biopsy specimens in standing sedated horses. *Am J Vet Res*. Mar 2008;69(3):431-9. doi:10.2460/ajvr.69.3.431	Experimental in vivo study<br>Experimental in vivo study<br>Experimental in vivo study	North America<br>North America<br>North America	2<br>7<br>13	Other<br>UG<br>GI	Experimental<br>Treatment<br>Experimental	Recumbency<br>Both<br>Standing	x<br>x<br>x	X
197. Bracamonte JL, Duke-Novakovski T. A pilot study evaluating laparoscopic closure of the nephrosplenic space using an endoscopic suturing device in standing horses. *Canadian Veterinary Journal-Revue Veterinaire Canadienne*. Jun 2016;57(6):651-654.	Prospective observational study	North America	4	Other	Experimental	Standing	x	
198. Bracamonte JL, Thomas KL. Laparoscopic cryptorchidectomy with a vessel-sealing device in dorsal recumbent horses: 43 cases. Article. *Veterinary Surgery*. 2017;46(4):559-565. doi:10.1111/vsu.12624	Retrospective case series	North America	43	UG	Treatment	Recumbency	x	X
199. Brink P, Schumacher J. Elevating the uterus (uteropexy) of five mares by laparoscopically imbricating the mesometrium. *Equine Veterinary Journal*. Nov 2010;42(8):675-679. doi:10.1111/j.2042-3306.2010.00069.x	Retrospective case series	North America	5	UG	Treatment	Standing	x	
200. Brommer H, Grinwis GCM, van Loon V, Ensink JM. Laparoscopic-assisted diagnosis of anomalous unilateral abdominal cryptorchidism. *Equine Veterinary Education*. Aug 2011;23(8):391-395. doi:10.1111/j.2042-3292.2011.00243.x	Case report	Europe	1	UG	Diagnostic	Standing	x	

201. Brugmans F, Deegen E. Laparoscopic surgical technique for repair of rectal and colonie tears in horses: An experimental study. Article. *Veterinary Surgery*. 2001;30(5):409-416. doi:10.1053/jvet.2001.25864	Experimental in vivo study	Europe	3	GI	Experimental	Recumbency	x	X
202. Burke MJ, Parente EJ. Prosthetic Mesh for Obliteration of the Nephrosplenic Space in Horses: 26 Clinical Cases. Article. *Veterinary Surgery*. 2016;45(2):201-207. doi:10.1111/vsu.12434	Retrospective case series	North America	26	Other	Preventive	Standing	x	X
203. Busschers E, Southwood LL, Parente EJ. Laparoscopic diagnosis and correction of a nephrosplenic entrapment of the large colon in a horse. *Equine Veterinary Education*. Mar 2007;19(2):60-63. doi:10.2746/095777307x181889	Case report	North America	1	GI	Diagnostic/tre atment	Standing	x	X
204. Butt TD, Wilson DG. Laparoscopic colopexy in a horse. *Can Vet J*. Jul 2003;44(7):586-8.	Case report	North America	1	GI	Preventive	Recumbency	x	X
205. Carluccio A, de Amicis I, Petrizzi L, et al. Laparoscopic drainage of an ovarian haematoma in a Martina Franca jenny (Equus asinus). Article. *Veterinarni Medicina*. 2020;65(8):358-363. doi:10.17221/29/2020-VETMED	Case report	Europe	1	UG	Treatment	Standing	x	
206. Caron JP, Brakenhoff J. Intracorporeal suture closure of the internal inguinal and vaginal rings in foals and horses. Article. *Veterinary Surgery*. 2008;37(2):126-131. doi:10.1111/j.1532-950X.2007.00366.x	Case series	North America	8	UG	Treatment/pre ventive	Recumbency	x	
207. Caron JP, Mehler SJ. Laparoscopic mesh incisional hernioplasty in five horses. Article. *Veterinary Surgery*. 2009;38(3):318-325. doi:10.1111/j.1532-950X.2009.00511.x	Case series	North America	5	Other	Treatment	Recumbency	x	
208. Cokelaere SM, Martens AMJG, Wiemer P. Laparoscopic ovariectomy in mares using a polyamide tie-rap. Article. *Veterinary Surgery*. 2005;34(6):651-656. doi:10.1111/j.1532-950X.2005.00101.x	Prospective observational study	Europe	10	UG	Experimental	Standing	x	X

209. Cokelaere SM, Martens A, Vanschandevijl K, Wilderjans H, Steenhaut M. Hand-assisted laparoscopic nephrectomy after initial ureterocystostomy in a Shire filly with left ureteral ectopia. *Vet Rec*. Sep 22 2007;161(12):424-7. doi:10.1136/vr.161.12.424	Case report	Europe	1	UG	Treatment	Standing		
210. Colbath AC, Hackett ES, Lesser CS, Hendrickson DA. Left paralumbar laparoscopic bilateral ovariectomy in mares. *Veterinary Surgery*. May 2017;46(4):574-579. doi:10.1111/vsu.12637	Prospective observational study	North America	20	UG	Experimental	Standing	x	
211. Collar EM, Duesterdieck-Zellmer KF, Huber MJ, Semevolos SA, Parker JE, Husby KA. Outcome of bilateral equid laparoscopic ovariectomies. Article. *Veterinary Surgery*. 2021;50(5):975-983. doi:10.1111/vsu.13651	Retrospective cohort study	North America	51	UG	Treatment/preventive	Standing	x	X
212. Comino F, Röcken M, Gorvy D. Standing laparoscopy combined with a conventional inguinal approach to treat extended septic funiculitis in 12 horses. *Vet Surg*. Apr 18 2022;doi:10.1111/vsu.13809	Retrospective case series	Multicentric	12	UG	Treatment	Standing	x	
213. Corsalini J, Gialletti R, Lotto E, Nannarone S. Laparoscopic Uteropexy (Mesometrium Imbrication) in Three Mares Using a Barbed Suture. *Journal of Equine Veterinary Science*. May 2016;40:102-105. doi:10.1016/j.jevs.2016.02.236	Prospective observational study	Europe	3	UG	Treatment	Standing	x	
214. Cribb NC, Arroyo LG, Boure L. Standing laparoscopic abdominal lavage using a suction-irrigation device in 2 horses with primary suppurative peritonitis. *Canadian Veterinary Journal-Revue Veterinaire Canadienne*. Apr 2021;62(4):397-402.	Case report	North America	2	Other	Treatment	Standing		X
215. Cribb NC, Boure LP. Laparoscopic Removal of a Large Abdominal Testicular Teratoma in a Standing Horse. *Veterinary Surgery*. Jan 2010;39(1):131-135. doi:10.1111/j.1532-950X.2009.00618.x	Case report	North America	1	UG	Treatment	Standing	x	
216. Cribb NC, Koenig J, Sorge U. Comparison of laparoscopic versus conventional open cryptorchidectomies on intraoperative and postoperative complications and duration of surgery, anesthesia, and hospital stay in horses. *Javma-Journal of the American Veterinary Medical Association*. Apr 2015;246(8):885-892. doi:10.2460/javma.246.8.885	Retrospective cohort study	North America	46	UG	Treatment	Standing		X

217. Cypher EE, Blackford J, Snowden RT, Sexton JA, Schumacher J. Surgical correction of entrapment of the large colon and caecum through a mesoduodenal rent with standing laparoscopic repair in a mare. *Equine Veterinary Education*. Apr 2020;32(4):185-188. doi:10.1111/eve.12941	Case report	North America	1	GI	Treatment	Standing	x	X
218. Daniel AJ, Easley JT, Story MR, Hendrickson DA, Hackett ES. Standing hand-assisted laparoscopic removal of large granulosa cell tumours in horses using a specimen retrieval bag and morcellator. *Equine Veterinary Education*. Oct 2015;27(10):505-509. doi:10.1111/eve.12374	Case report	North America	3	UG	Treatment	Standing	x	
219. Daniel A, McCue P, Ferris R, Miller C, Leise B. Bilateral ovarian leiomyoma treated by standing laparoscopic ovariectomy. *Equine Veterinary Education*. Oct 2015;27(10):510-514. doi:10.1111/eve.12438	Case report	North America	1	UG	Treatment	Standing	x	
220. Davis EW. Laparoscopic cryptorchidectomy in standing horses. *Veterinary Surgery*. Jul-Aug 1997;26(4):326-331. doi:10.1111/j.1532-950X.1997.tb01505.x	Prospective clinical study	North America	11	UG	Treatment	Standing	x	
221. de Fourmestraux C, Geffroy O, Siliart B, Albaric O, Tessier C. Evaluation of success rate of laparoscopic castration without orchidectomy in 32 mature horses. *Equine Veterinary Education*. Jan 2014;26(1):34-39. doi:10.1111/eve.12089	Retrospective case series	Europe	32	UG	Treatment	Standing		X
222. Delcazo M, Geburek F, Kohler K, Rocken M, Theiss F. Laparoscopic resection of an exostosis of the os pubis in a horse. *Veterinary Surgery*. Apr 2020;49(3):614-620. doi:10.1111/vsu.13349	Case report	Europe	1	Other	Treatment	Standing	x	
223. Delli-Rocili MM, Cribb NC, Trout DR, Thomason JJ, Valverde A. Effectiveness of a paraverterbral nerve block versus local portal blocks for laparoscopic closure of the nephrosplenic space: A pilot study. *Veterinary Surgery*. Jul 2020;49(5):1007-1014. doi:10.1111/vsu.13452	Randomized clinical trial	North America	12	Other	Experimental	Standing		
224. Delling U, Howard RD, Pleasant RS, Lanz OI. Hand-assisted laparoscopic ovariohysterectomy in the mare. *Vet Surg*. Sep-Oct 2004;33(5):487-94. doi:10.1111/j.1532-950x.2004.04063.x	Experimental in vivo study	North America	8	UG	Experimental	Recumbency	x	

225. Delling U, Stoebe S, Brehm W. Hand-assisted laparoscopic adhesiolysis of extensive small intestinal adhesions in a mare after breeding injury. *Equine Veterinary Education*. Nov 2012;24(11):545-551. doi:10.1111/j.2042-3292.2011.00333.x	Case report	Europe	1	GI	Treatment	Standing	x	
226. Desmaizières LM, Martinot S, Lepage OM, Bareiss E, Cadoré JL. Complications associated with cannula insertion techniques used for laparoscopy in standing horses. Article. *Veterinary Surgery*. 2003;32(6):501-506. doi:10.1111/j.1532-950X.2003.00501.x	Retrospective cohort study	Europe	40	Other	Diagnostic/treatment	Standing	x	X
227. Devick IF, Hendrickson DA. Left paralumbar fossa approach combined with mesocolon fenestration for bilateral equine ovariectomy. *Vet Surg*. Jul 2019;48(5):735-741. doi:10.1111/vsu.13166	Experimental in vivo study	North America	5	UG	Experimental	Standing	x	
228. Devick IF, Leise BS, Rao S, Hendrickson DA. Evaluation of post-operative pain after active desufflation at completion of laparoscopy in mares undergoing ovariectomy. *Canadian Veterinary Journal-Revue Veterinaire Canadienne*. Mar 2018;59(3):261-266.	Prospective observational study	North America	38	UG	Treatment	Standing	x	
229. Diekstall M, Rijkenhuizen A. Mesorectal hematoma associated with colic and caudal neurological signs in a horse. *Pferdeheilkunde*. May-Jun 2018;34(3):232-236. doi:10.21836/pem20180303	Case report	Europe	1	Other	Diagnostic	Standing	x	X
230. Diekstall M, Rohde C, Rijkenhuizen ABM. Post-partum uterine rupture: Standing repair in three mares using a laparoscopic technique. *Equine Veterinary Education*. Jun 2020;32(6):319-324. doi:10.1111/eve.13001	Prospective observational study	Europe	3	UG	Treatment	Standing	x	
231. Düsterdieck KF, Pleasant RS, Lanz OI, Saunders G, Howard RD. Evaluation of the harmonic scalpel for laparoscopic bilateral ovariectomy in standing horses. *Vet Surg*. May-Jun 2003;32(3):242-50. doi:10.1053/jvet.2003.50022	Experimental in vivo study	North America	8	UG	Experimental	Standing	x	X
232. Easley JT, McGilvray KC, Hendrickson DA, Bruemmer J, Hackett ES. Vessel sealer and divider instrument temperature during laparoscopic ovariectomy in horses. Article. *Veterinary Surgery*. 2018;47:O26-O31. doi:10.1111/vsu.12755	Prospective observational study	North America	15	UG	Experimental	Standing	x	X

233. El-Sherif MW, El-Khamary AN, Abdel-Wahed RE. Lateral versus dorsal recumbent laparoscopic cryptorchidectomy and castration in horses. *Online Journal of Veterinary Research*. 2017;21(8):523-530.	Prospective case-control	Asia	20	UG	Treatment	Recumbency		
234. Epstein KL, Parente EJ. Laparoscopic obliteration of the nephrosplenic space using polypropylene mesh in five horses. *Veterinary Surgery*. Jul 2006;35(5):431-437. doi:10.1111/j.1532-950X.2006.00171.x	Experimental in vivo study	North America	5	Other	Experimental	Standing		X
235. Farstvedt E, Hendrickson D. Laparoscopic closure of the nephrosplenic space for prevention of recurrent nephrosplenic entrapment of the ascending colon. *Veterinary Surgery*. Nov-Dec 2005;34(6):642-645. doi:10.1111/j.1532-950X.2005.00099.x	Retrospective case series	North America	10	Other	Preventive	Standing	x	
236. Fernández-Parra R, Losada-Floriano A, Zilberstein L, Bourzac C. Iatrogenic pneumothorax-induced heart murmur during standing laparoscopy in a 3-year-old horse. Article. *Equine Veterinary Education*. 2022;doi:10.1111/eve.13641	Case report	Europe	1	Other	Preventive	Standing	x	X
237. Finley CJ, Fischer AT. Removal of equine cryptorchid testes through an enlarged umbilical portal in dorsally recumbent horses after intra-abdominal laparoscopic castration. *Equine Veterinary Journal*. doi:10.1111/evj.13483	Retrospective case series	North America	79	UG	Treatment	Recumbency	x	
238. Gablehouse KB, Cary J, Farnsworth K, Ragle CA. Standing laparoscopic-assisted vaginal ovariohysterectomy in a mare. *Equine Veterinary Education*. Jun 2009;21(6):303-306. doi:10.2746/095777309x446603	Case report	North America	1	UG	Treatment	Standing	x	X
239. Gandini M, Giusto G, Caramello V, Comino F, Rosso A. Single-port laparoscopic incisional hernia repair in a horse. *Equine Veterinary Education*. Jul 2017;29(7):362-366. doi:10.1111/eve.12547	Case report	Europe	1	Other	Treatment	Recumbency	x	X
240. Gandini M, Labate F, Rosso A, Giusto G. Strangulating obstruction of the small intestine by a fibrous band originating on the nephrosplenic ligament. *Equine Veterinary Education*. May 2021;33(5):E146-E148. doi:10.1111/eve.13206	Case report	Europe	1	GI	Treatment	Standing	x	X

241. Gandini M, Nannarone S, Giusto G, et al. Laparoscopic nephrosplenic space ablation with barbed suture in eight horses. *Javma-Journal of the American Veterinary Medical Association*. Feb 2017;250(4):431-436. doi:10.2460/javma.250.4.431	Case series	Europe	8	Other	Preventive	Standing		
242. Gardner AK, Santschi EM, Aeffner F, Pigott JH, Russell DS. Testicular ischaemic necrosis as a cause of equine cryptorchidism. Article. *Equine Veterinary Education*. 2017;29(6):314-317. doi:10.1111/eve.12544	Case report	North America	1	UG	Treatment	Recumbency	x	
243. Gialletti R, Nannarone S, Gandini M, et al. Comparison of Mesh and Barbed Suture for Laparoscopic Nephrosplenic Space Ablation in Horses. *Animals*. Apr 2021;11(4)1096. doi:10.3390/ani11041096	Retrospective case-control	Europe	28	Other	Preventive	Standing	x	
244. Goodin JT, Rodgerson DH, Gomez JH. Standing Hand-Assisted Laparoscopic Ovariectomy in 65 Mares. Article. *Veterinary Surgery*. 2011;40(1):90-92. doi:10.1111/j.1532-950X.2010.00771.x	Case series	North America	65	UG	Treatment	Standing		X
245. Goto A, Tagami M, Kato F, et al. Equine nonneoplastic abnormal ovary in a draft mare with high serum anti-Müllerian hormone: a case study. *Journal of Equine Science*. 2021;32(4):147-151. doi:10.1294/jes.32.147	Case report	Asia	1	UG	Treatment	Standing	x	X
246. Gottschalk RD, Berg SSvd. Standing laparoscopically-aided ovariectomy in mares. *Journal of the South African Veterinary Association*. 1997;68(3):102-104.	Case series	Africa	13	UG	Treatment	Standing		
247. Gracia-Calvo LA, Duque J, Balao da Silva C, Ezquerra J, Ortega-Ferrusola C. Testicular perfusion after standing laparoscopic peritoneal flap hernioplasty in stallions. Article. *Theriogenology*. 2015;84(5):797-804. doi:10.1016/j.theriogenology.2015.05.014	Case series	Europe	6	UG	Treatment	Standing		X
248. Gracia-Calvo LA, Ezquerra LJ, Martín-Cuervo M, et al. Standing laparoscopic peritoneal flap hernioplasty of the vaginal rings does not modify the sperm production and motility characteristics in intact male horses. *Reproduction in Domestic Animals*. 2014;49(6):1043-1048. doi:10.1111/rda.12434	Case series	Europe	6	UG	Preventive	Standing		

249. Gracia-Calvo LA, Ezquerra LJ, Ortega-Ferrusola C, et al. Histological findings in equine testes one year after standing laparoscopic peritoneal flap ernioplasty. Article. *Veterinary Record*. 2016;178(18):450. doi:10.1136/vr.103236	Case series	Europe	10	Other	Preventive	Standing		
250. Gracia-Calvo L, Martín-Cuervo M, Jiménez J, et al. Development of a technique for standing hand-assisted laparoscopic splenectomy in five horses. Article. *Australian Veterinary Journal*. 2015;93(6):183-188. doi:10.1111/avj.12326	Case series	Europe	5	Other	Experimental	Standing	x	
251. Hand R, Rakestraw P, Taylor T. Evaluation of a vessel-sealing device for use in laparoscopic ovariectomy in mares. *Vet Surg*. May-Jun 2002;31(3):240-4. doi:10.1053/jvet.2002.33482	Prospective clinical study	North America	13	UG	Preventive	Standing	x	
252. Hanrath M, Rodgerson DH. Laparoscopic cryptorchidectomy using electrosurgical instrumentation in standing horses. Article. *Veterinary Surgery*. 2002;31(2):117-124. doi:10.1053/jvet.31049	Retrospective clinical study	North America	10	UG	Treatment	Standing	x	
253. Hanson CA, Galuppo LD. Bilateral laparoscopic ovariectomy in standing mares: 22 Cases. Article. *Veterinary Surgery*. 1999;28(2):106-112. doi:10.1053/jvet.1999.0106	Prospective clinical study	North America	22	UG	Treatment	Standing	x	
254. Hendrickson DA, Wilson DG. Laparoscopic cryptorchid castration in standing horses. *Veterinary Surgery*. Jul-Aug 1997;26(4):335-339. doi:10.1111/j.1532-950X.1997.tb01507.x	Prospective clinical study	North America	8	UG	Diagnostic/Treatment	Standing	x	X
255. Hilton HG, Aleman M, Maher O, Peterson TS, Whitcomb MB, Galuppo LD. Hand-assisted laparoscopic nephrectomy in a standing horse for the management of renal cell carcinoma. *Equine Veterinary Education*. May 2008;20(5):239-244. doi:10.2746/095777308x295783	Case report	North America	1	Other	Treatment	Standing	x	X
256. Holmes JM, Nath LC, Muurlink MA. Laparoscopic cauterisation of the testicular arteries to manage haemoperitoneum in a gelding. *Equine Veterinary Education*. Jun 2013;25(6):297-300. doi:10.1111/j.2042-3292.2012.00416.x	Case report	Australia	1	UG	Treatment	Standing		X

257. Hoogmoed LMv, Galuppo LD. Laparoscopic ovariectomy using the Endo-GIA stapling device and Endo-Catch pouches and evaluation of analgesic efficacy of epidural morphine sulfate in 10 mares. *Veterinary Surgery*. 2005;34(6):646-650. doi:10.1111/j.1532-950X.2005.00100.x	Randomized clinical trial	North America	10	UG	Experimental	Standing	x	
258. Hubert JD, Burba DJ, Moore RM. Evaluation of a vessel-sealing device for laparoscopic granulosa cell tumor removal in standing mares. *Vet Surg*. Jun 2006;35(4):324-9. doi:10.1111/j.1532-950X.2006.00151.x	Retrospective case series	North America	8	UG	Treatment	Standing	x	X
259. Jones ARE, Ragle CA, Anderson D, Scott C. Laparoscopic evaluation of the small intestine in the standing horse: Technique and effects*. Article. *Veterinary Surgery*. 2017;46(6):812-820. doi:10.1111/vsu.12664	Prospective pilot study	North America	5	GI	Experimental	Standing	x	
260. Jones ARE, Ragle CA, Huggons NA, Tibary AA. Bilateral ovariectomy as a treatment for chronic pyometra in four horses. *Equine Veterinary Education*. Oct 2020;32(10):E189-E193. doi:10.1111/eve.13167	Case series	North America	4	UG	Treatment	Standing		
261. Kadic DTN, Bonilla AG. A two-step ovariohysterectomy with unilateral left flank laparoscopic assistance in a Quarter Horse mare. *Equine Veterinary Education*. Oct 2020;32(10):E199-E202. doi:10.1111/eve.13131	Case report	North America	1	UG	Treatment	Standing	x	
262. Kambayashi Y, Tsuzuki N, Seo J, et al. Evaluation of single-incision laparoscopic ovariectomy in standing mares. *Journal of Equine Veterinary Science*. 2014;34(3):446-450. doi:10.1016/j.jevs.2013.07.006	Case series	Asia	5	UG	Treatment	Standing	x	X
263. Kamus L.P. Ruzickova,P. Piat,P. Trencart,A. BonillaUse of barbed suture for thoracoscopic repair of diaphragmatic hernias: Three cases. Equine Vet edu. 2022	Case series	Europe	4	Other	Treatment	Standing		X
264. Keoughan CG, Rodgerson DH, Brown MP. Hand-assisted laparoscopic left nephrectomy in standing horses. *Vet Surg*. May-Jun 2003;32(3):206-12. doi:10.1053/jvet.2003.50028	Prospective clinical study	North America	8	UG	Treatment	Standing		

265. Klohnen A, Wilson DG. Laparoscopic repair of scrotal hernia in two foals. *Vet Surg*. Sep-Oct 1996;25(5):414-16. doi:10.1111/j.1532-950x.1996.tb01437.x	Case report	North America	2	GI	Treatment	Recumbency	x	
266. Koch DW, Easley JT, Hatzel JN, et al. Prospective randomized investigation of topical anesthesia during unilateral laparoscopic ovariectomy in horses. *Vet Surg*. Jun 2020;49 Suppl 1:O54-o59. doi:10.1111/vsu.13264	Randomized clinical trial	North America	15	UG	Experimental	Standing	x	
267. Köllmann M, Rötting A, Heberling A, Sieme H. Laparoscopic techniques for investigating the equine oviduct. *Equine Vet J*. Jan 2011;43(1):106-11. doi:10.1111/j.2042-3306.2010.00143.x	Prospective clinical study	Europe	10	UG	Experimental	Standing	x	
268. Kummer M, Theiss F, Jackson M, Fürst A. Evaluation of a Motorized Morcellator for Laparoscopic Removal of Granulosa-Theca Cell Tumors in Standing Mares. Article. *Veterinary Surgery*. 2010;39(5):649-653. doi:10.1111/j.1532-950X.2010.00688.x	Case series	Europe	7	UG	Treatment	Standing	x	
269. Lacitignola L, Imperante A, De Siena R, et al. Wound Retractor Laparoscopic Port System for Standing Laparoscopic Cryptorchidectomy in the Horse: A Case Report. *Journal of Equine Veterinary Science*. Sep 2020;92103168. doi:10.1016/j.jevs.2020.103168	Case report	Europe	1	UG	Treatment	Standing	x	
270. Lawless SP, Moorman VJ, Hendrickson DA, Mama KR. Comparison of sedation quality and safety of detomidine and romifidine as a continuous rate infusion for standing elective laparoscopic ovariectomy in mares. *Vet Surg*. Jul 2021;50(5):990-998. doi:10.1111/vsu.13627	Prospective clinical study	North America	18	UG	Experimental	Standing		X
271. Lloyd D, Walmsley JP, Greet TRC, Payne RJ, Newton JR, Phillips TJ. Electrosurgery as the sole means of haemostasis during the laparoscopic removal of pathologically enlarged ovaries in mares: a report of 55 cases. *Equine Veterinary Journal*. May 2007;39(3):210-214. doi:10.2746/042516407x17116522	Case series	Europe	55	UG	Treatment	Standing		
272. Lund CM, Ragle CA, Dylan Lutter J, Farnsworth KD. Use of a motorized morcellator for elective bilateral laparoscopic ovariectomy in standing equids: 30 cases (2007-2013). Article. *Journal of the American Veterinary Medical Association*. 2014;244(10):1191-1197. doi:10.2460/javma.244.10.1191	Retrospective case series	North America	30	UG	Treatment	Standing		

273. Lund CM, Ragle CA, Lutter JD. Laparoscopic removal of a bladder urolith in a standing horse. *J Am Vet Med Assoc*. Nov 1 2013;243(9):1323-8. doi:10.2460/javma.243.9.1323	Case report	North America	1	UG	Treatment	Standing		X
274. Mariën T. Standing laparoscopic herniorrhaphy in stallions using cylindrical polypropylene mesh prosthesis. *Equine Vet J*. Jan 2001;33(1):91-6. doi:10.2746/042516401776767476	Case series	Europe	9	GI	Treatment	Standing	x	
