# Supplementary material for: Occurrence and Definitions of Intra and Postoperative Complications Related to Laparoscopy in Equids: A Scoping Review
Source: Vet Sci. 2022 Oct 17;9(10):577. doi: 10.3390/vetsci9100577 (PMC9609183; doi:10.3390/vetsci9100577)
Supplement: Supplementary file 1 [file vetsci-09-00577-s001.zip › Supplementary material 3.pdf]

Table S1. Definition for signs of pain or discomfort reported in 1 out of 5 articles which reported signs of pain or discomfort as an intraoperative complication.

Definition	References
Lifting a hind limb, looking back at the flank	127

Table S2. Definition for poor portal placement reported in 1 out of 2 articles which reported poor portal placement as an intraoperative complication.

Definition	References
Each observation was scored from 1 (absence of gross pain, head above withers, ears forward, alert, moves freely, freely lift feet when asked, responds to door opening, approach, and reaches for grain) to 4 (continuous signs of gross pain, head below withers, ears back, does not move, located in the middle or facing the back of the stall, unwilling to lift feet, no response to door opening, observer approaching, or offer of grain).	106

Table S3. Heamorrhage from mesovarium reported in articles on laparoscopic ovariectomy where vessel sealing devices were used for hemostasis

	**Vessel sealing devices**					
	**N of cases with hemorrhage**	**N of cases in articles reporting hemorrhage**	**Relative incidence**	**N of cases of all articles reporting ovariectomy**	**Cumulative incidence (on ovariectomy cases)**	**Cumulative incidence (on 2321 cases)**
1995-1999	0	0	0	0	0	0
2000-2004	11	21	52,4	64	17,2	0,5
2005-2009	1	12	8,3	81	1,2	0,0
2010-2014	6	49	12,2	267	2,2	0,3
2015-2019	0	0	0,0	170	0,0	0,0
2020-2021	19	44	43,2	145	13,1	0,8
**TOTAL**	37	126	29,4	727	5,1	1,6

Table S4. Heamorrhage from mesovarium reported in articles on laparoscopic ovariectomy where other devices (ligatures, surgical staplers) were used for hemostasis

	**Other methods of hemostasis**					
	**N of cases with hemorrhage**	**N of cases in articles reporting hemorrhage**	**Relative incidence**	**N of cases of all articles reporting ovariectomy**	**Cumulative incidence (on ovariectomy cases)**	**Cumulative incidence (on 2321 cases)**
1995-1999	1	15	6,7	76	1,3	0
2000-2004	5	18	27,8	28	17,9	0,2
2005-2009	4	26	15,4	267	1,5	0,2
2010-2014	18	74	24,3	124	14,5	0,8
2015-2019	0	0	0,0	87	0,0	0,0
2020-2021	1	79	0,0	79	1,3	0,0
**TOTAL**	29	212	13,7	661	4,4	1,2

Table S5. Definition for failure of the first procedure reported in 1 article out of 2 articles which reported failure of the first procedure as a postoperative complication.

Definition	Reference
Failure of the suture/mesh to fully ablate the NSS	61

Table S6. Definition for anorexia reported in 1 out of 7 articles which reported anorexia as a postoperative complication.

Definition	Reference
Decrease in appetite for 12-48 h	71

Table S7. Definition for postoperative pain in 11 out of 54 articles which reported postoperative pain as a postoperative complication.

Definition	Reference
Composite pain score	46,77
Postoperative pain was scored using a VAS and 13 category, multifactorial composite pain scale at 1 h, and every 6 h, to 24 h postoperatively	88
Pain behaviors were assessed during the first two post-operative days using a numerical rating scale	94
VAS scale	146,147
Inappetance, pawing, flank watching and lying in sternal recumbency	20
High heart rate, recumbency	42
60 bpm, sweating, lying down, decreased intestinal motility	47
Lying down, pawing	126
Wanting to lie down immediately after being returned in stall	38

Table S8. Definition for incisional complications in 8 out of 106 articles which reported incisional complications as a postoperative complication.

Definition	Reference
Incisional drainage	29
Moderate subcutaneous edema and pain at palpation that regressed with NSAIDs therapy	108
Swelling area diagnosed ultrasonographically	11
Pocket of serosanguineous s fluid near incision site	68
Incisional complications were defined as any record of serous or purulent wound discharge or parting of wound edges	89
Surgical site cosmesis (depression, thickening, white hairs on surgical portals sites)	161
Sensitivity around the laparoscopy portal incisions postoperatively	74,109

Table S9. Definition for decrease of fecal output in 1 out of 3 articles which reported decrease of fecal output as a postoperative complication.

Definition	Reference
Fecal output (piles of manure/12 h and piles of manure/24 h) was monitored postoperatively and compared to preoperative fecal output (12 h before and 24 h before for mares that were hospitalized this long, and for which this number was available). Fecal output was manually recorded hourly (preperatively and postoperatively) and tallied for each 12 h period	88

Table S10. Definition for haemoabdomen in 1 out of 3 articles which reported haemoabdomen as a postoperative complication.

Definition	Reference
Echogenic, swirling fluid within the abdominal cavity consistent with blood was identified on abdominal ultrasonography	55

Table S11. Definition for AST/AMY alteration in 1 article which reported AST/AMY alteration as a postoperative complication.

Definition	Reference
There was a significant increase in AST (P ¼.001) and AMY (P ¼.017) from baseline (day 0) on day 1 after surgery and AST was also beyond the laboratory reference interval (144–350 U/L) on day 1	99

Table S12. Definition for piroplasmosis in 2 out of 2 articles which reported piroplasmosis as a postoperative complication.

Definition	Reference
Babesia equi infection diagnosed on haematology	64
1 Case: fever > 39°C and icteric mucous membranes, A blood sample was collected for haematological and biochemical analyses, which indicated decreased packed cell volume (28%, reference range 30–45%), haemolysis and slightly increased unconjugated bilirubin (3.9 mg/dL, reference range 0.2–2 mg/dL). Peripheral blood cytology confirmed the presence of Babesia merozoites in the erythrocytes. 1 Case: igns of piroplasmosis (fever >39°C and icteric mucous membranes). The serum antibodies titre for Theileria equi was 1/340 (indirect immunofluorescence antibody test limit 1/80); however, merozoites were not observed within the erythrocytes.	68

Table S13. Definition for phlebitis in 1 out of 2 articles which reported phlebitis as a postoperative complication.

Definition	Reference
Enlarged painful mass around the indwelling catheter and ultrasonographic findings consistent with thrombus formation.	32
